# Diagnostic classification of childhood cancer using multiscale transcriptomics

**DOI:** 10.1038/s41591-023-02221-x

**Published:** 2023-03-17

**Authors:** Federico Comitani, Joshua O. Nash, Sarah Cohen-Gogo, Astra I. Chang, Timmy T. Wen, Anant Maheshwari, Bipasha Goyal, Earvin S. Tio, Kevin Tabatabaei, Chelsea Mayoh, Regis Zhao, Ben Ho, Ledia Brunga, John E. G. Lawrence, Petra Balogh, Adrienne M. Flanagan, Sarah Teichmann, Annie Huang, Vijay Ramaswamy, Johann Hitzler, Jonathan D. Wasserman, Rebecca A. Gladdy, Brendan C. Dickson, Uri Tabori, Mark J. Cowley, Sam Behjati, David Malkin, Anita Villani, Meredith S. Irwin, Adam Shlien

**Affiliations:** 1 Program in Genetics and Genome Biology, The Hospital for Sick Children, Toronto, ON Canada; 2 Laboratory of Medicine and Pathobiology, University of Toronto, Toronto, ON Canada; 3 Department of Paediatrics, The Hospital for Sick Children and University of Toronto, Toronto, ON Canada; 4 Children’s Cancer Institute, Lowy Cancer Research Centre, UNSW Sydney, Sydney, NSW Australia; 5 School of Clinical Medicine, UNSW Sydney, Sydney, NSW Australia; 6 The Arthur and Sonia Labatt Brain Tumour Research Centre, The Hospital for Sick Children, Toronto, ON Canada; 7 Wellcome Sanger Institute, Hinxton, UK; 8 Department of Cellular and Molecular Pathology, Royal National Orthopaedic Hospital, Brockley Hill, Stanmore, UK; 9 Research Department of Pathology, University College London Cancer Institute, London, UK; 10 Medical Biophysics, University of Toronto, Toronto, ON Canada; 11 Program in Developmental and Stem Cell Biology, The Hospital for Sick Children Research Institute, Toronto, ON Canada; 12 Department of Surgical Oncology, Princess Margaret Cancer Centre/Mount Sinai Hospital, Toronto, ON Canada; 13 Department of Surgery, University of Toronto, Toronto, ON Canada; 14 Lunenfeld-Tanenbaum Research Institute, Sinai Health System, Toronto, ON Canada; 15 Department of Pathology and Laboratory Medicine, Mount Sinai Hospital, University of Toronto, Toronto, ON Canada; 16 Cambridge University Hospitals NHS Foundation Trust, Cambridge, UK; 17 Department of Paediatrics, University of Cambridge, Cambridge, UK

**Keywords:** Paediatric cancer, Machine learning, Cancer genomics, Gene expression

## Abstract

The causes of pediatric cancers’ distinctiveness compared to adult-onset tumors of the same type are not completely clear and not fully explained by their genomes. In this study, we used an optimized multilevel RNA clustering approach to derive molecular definitions for most childhood cancers. Applying this method to 13,313 transcriptomes, we constructed a pediatric cancer atlas to explore age-associated changes. Tumor entities were sometimes unexpectedly grouped due to common lineages, drivers or stemness profiles. Some established entities were divided into subgroups that predicted outcome better than current diagnostic approaches. These definitions account for inter-tumoral and intra-tumoral heterogeneity and have the potential of enabling reproducible, quantifiable diagnostics. As a whole, childhood tumors had more transcriptional diversity than adult tumors, maintaining greater expression flexibility. To apply these insights, we designed an ensemble convolutional neural network classifier. We show that this tool was able to match or clarify the diagnosis for 85% of childhood tumors in a prospective cohort. If further validated, this framework could be extended to derive molecular definitions for all cancer types.

## Abstract (web summary)

A new multilevel clustering approach applied retrospectively to 13,000 transcriptomes of different tumors reveals a new diagnostic classification of childhood cancers, in some cases allowing a better prediction of disease outcomes.

## Main

Over 400,000 childhood cancers are diagnosed per year worldwide^1^. Compared to adult cancers, childhood tumors are more likely to emerge from embryonic tissue and impact different cell types^2–5^. Most adult extracranial solid tumors are carcinomas, whereas mesodermal and embryonal tumors are more frequent in children^6^. One-third of childhood cancers are leukemias, which are proportionally not as common in adults. The same is true of neuroblastoma, a heterogeneous cancer ranging from a spontaneously regressing form in infants to a malignant progressing entity in older children and adolescents and rarely found in adults^2,3^.

Currently, no comprehensive molecular assay can aid in the diagnosis of all pediatric cancers. Genome sequencing can reveal the tumor’s history, including mutations preceding its malignant transformation^7^, and can be disconnected from the tumor’s current phenotype. On the other hand, RNA sequencing (RNA-seq) is reflective of the tumor’s ongoing expression program and can differentiate tumors independent of genomic origin^8^. Because a critical number of childhood tumor transcriptomes are or will soon be available^9^, RNA-seq has the potential to become a standalone ‘universal diagnostic assay’.

Most transcriptome-based classifiers are fully supervised tools, reliant on the tumors’ pre-existing labels without allowing for much phenotypic variability. However, intra-tumoral transcriptional differences can be so pronounced that they result in both favorable and poor prognostic signatures within the same tumor^10,11^. Stromal or immune infiltration also adds to this diversity^12,13^. In this Article, we identify features that define the unique gene expression profiles of childhood cancers compared to adult neoplasms. By incorporating measures of transcriptional entropy, we calculate their heterogeneity at multiple levels, both between subtly different tumor types as well as across major classes of cancer. Far from being ‘quiet’^5,14^, as suggested by DNA analyses^15^, childhood tumors have more transcriptional diversity, both between and within tumor types, than most adult cancers. Accounting for this variability can be leveraged to improve the tools used to diagnose childhood cancer. To this end, we built RACCOON (Resolution-Adaptive Coarse-to-fine Clusters OptimizatiON), a scale-adaptive clustering approach for the unsupervised classification of tumor subtypes using RNA-seq. It yielded an atlas of 455 tumor and normal classes when applied to a cohort of 13,313 samples, which were organized into a hierarchical tree based on their expression similarities. We also designed a classifier for childhood cancer, called OTTER (Oncologic TranscripTome Expression Recognition), an ensemble of convolutional neural networks (CNNs) targeting this extensive hierarchy. It is unique in scope and performs robustly even when using a fraction of the RNA-seq data (that is, a few million reads). When applied to a held-out cohort, OTTER was concordant with clinical pathology diagnoses in 82% of patients, helping to clarify the diagnosis for an additional 7% of the cases. Collectively, this work both defines the transcriptional distinctiveness of childhood cancer and uses this to validate a novel, pan-cancer diagnostic assay.

## Results

### RACCOON provides an accurate classification of human cancer

**Fig. 1:**
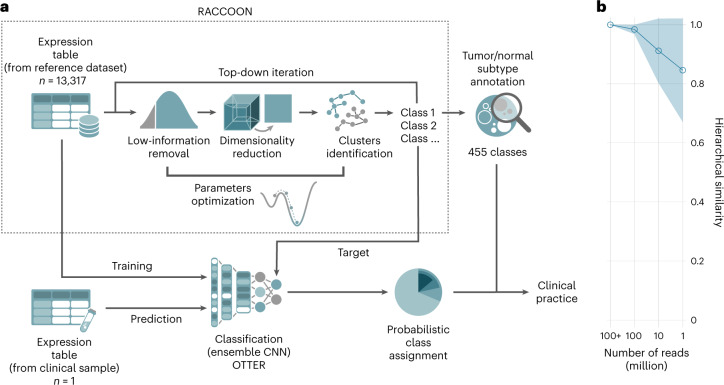
A platform for clustering and classification of RNA-seq data. **a**, Schematic representation of the steps involved in our RNA-seq tumor subtype identification protocol. We first built an extensive reference hierarchy of tumor and normal subtypes using RACCOON, a novel scale-adaptive clustering framework. This hierarchy was then used as a target for OTTER, an ensemble of CNN classifiers, which can be employed to identify multiple tumor and normal tissue components in samples from clinical practice. **b**, OTTER performance as a function of the number of sequenced reads. This is quantified as the hierarchical similarity (Methods) between the prediction probabilities obtained on subsampled data and the original sample (>1 × 10^8^ reads). Values are presented as mean and standard deviation of six tumor samples with reads randomly subsampled five times each. Expression counts were obtained with a STAR + RSEM pipeline.

To develop molecular definitions of childhood cancers, we designed a method that reduces the complexity of RNA-sequenced tumors and then groups them into hierarchically organized clusters (Fig. 1a and Methods). This was done in a way that would enable a deeper exploration of the transcriptional differences between and within tumor classes and would facilitate the discovery of new tumor subtypes. The key technical innovations used in our method, called RACCOON, are as follows: (1) the automatic optimization of parameters—for low-information filtering, dimensionality reduction and cluster identification—removing the need for tumor-type-specific expertise when choosing these parameters and (2) the iterative top-down building of hierarchies in a way that is scale and dataset independent.

Using this approach on a reference set of 2,178 childhood and 9,400 adult tumors, as well as 1,735 non-neoplastic samples^16–19^ (Methods), revealed a hierarchy of 455 clusters (or classes), representing 406 types of cancer. Of these, 69 classes are pediatric, and 49 classes are of non-neoplastic, normal tissue. As expected, the silhouette coefficient, which quantifies how distinct clusters are one from another, was high across tumor types and subtypes (Supplementary Fig. 1).

We then built a set of mono-dimensional CNNs^20^ to match individual patients to the tumor classes with high accuracy. The networks required for tumor classification were broad and shallow (in comparison to image classification, which requires extremely deep CNNs), in line with previous observations^21^. This suggests that a large number of patterns need to be evaluated to accurately diagnose cancer but that there is limited complexity of interactions between the genes involved.

**Extended Data Fig. 1:**
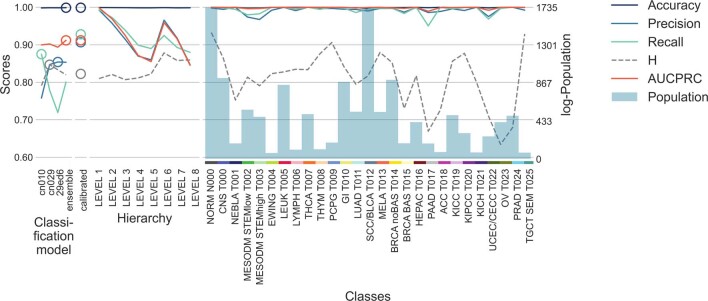
Classifier scores by model, level, and transcriptional family. Validation scores obtained by the randomized 5 × 5-fold validation. These include accuracy (dark blue), area under the curve precision-recall (AUCPR, orange), precision (blue) recall (green) and hierarchical similarity H (dashed grey). All averaged scores are calculated as micro (m) averages. The total reference population of each class is also shown as shaded bars (blue). These scores are shown per model in the ensemble (**a**), where the best model for each score is marked by a circle, per level (**b**), and per class, including only classes at the first level of the hierarchy (**c**). The ensemble reaches 0.955 5 × 5-fold validation median MAUCPR and 0.997 MF1 after calibration. In the per-level breakdown, MAUCPR goes from 0.998 for major tumor type classes, to 0.845 at its minimum for classes at the deepest level. The accuracy scores improve around level 7, due to the asymmetrical structure of the classes’ hierarchy. Only CNS and leukemias have subtypes extending beyond that level.

The top three performing CNNs were integrated into an ensemble, OTTER, which achieved higher scores across all metrics than any single model (Extended Data Fig. 1) and published classifiers. Because tumors can contain multiple distinct cell populations, as well as intermixed stroma or immune infiltrate, the classifier was designed to be both multiclass and multilabel. As such, OTTER reports the probabilities that a tumor belongs to a class, as well as its offspring classes, giving a refined view of a tumor’s subtype within a specific tumor ‘lineage’. A patient’s cancer can also match multiple labels depending on, for example, the admixture of distinct cell populations found within the same tissue.

OTTER maintains high performance across all cancer types (Supplementary Fig. 2) as well as in the presence of multiple tumor mixtures, high normal contamination or technical noise (Supplementary Figs. 3–5). More importantly, tumor matching is robust even with very shallow sequencing (Fig. 1b). Using only a few million reads, OTTER can output highly consistent predictions in just a few minutes (Supplementary Fig. 6).

### Pediatric tumors: many subtypes, few cell-of-origin groups

**Fig. 2:**
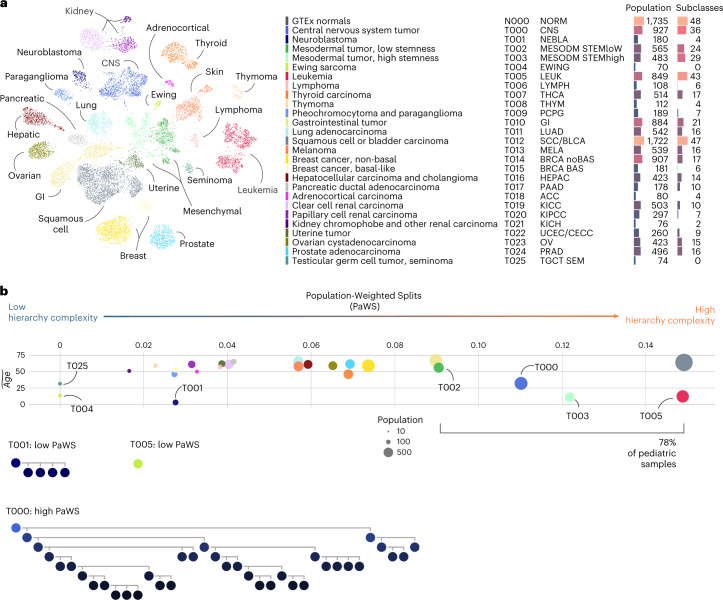
Transcriptional atlas of cancer. **a**, Two-dimensional UMAP projection of gene expression counts representative of the first level of hierarchy obtained with RACCOON on our reference tumor dataset, with classes drawn with different colors. For simplicity, only tumor subtypes are included in this representation. On the right is a legend including 27 transcriptional families identified at the first level of the hierarchy. For each class, full name, identification code, short name, number of member samples and number of subclasses are reported. **b**, PaWS scores measured on each of the main 27 transcriptional families. The marker size is proportional to the population of the cluster. This score quantifies the relative number of offspring for each class, adjusted by the population bias (see Methods for a formal definition). At the bottom are exemplary class hierarchical branches of three tumor types: neuroblastoma, ES and CNS tumors.

**Extended Data Fig. 2:**
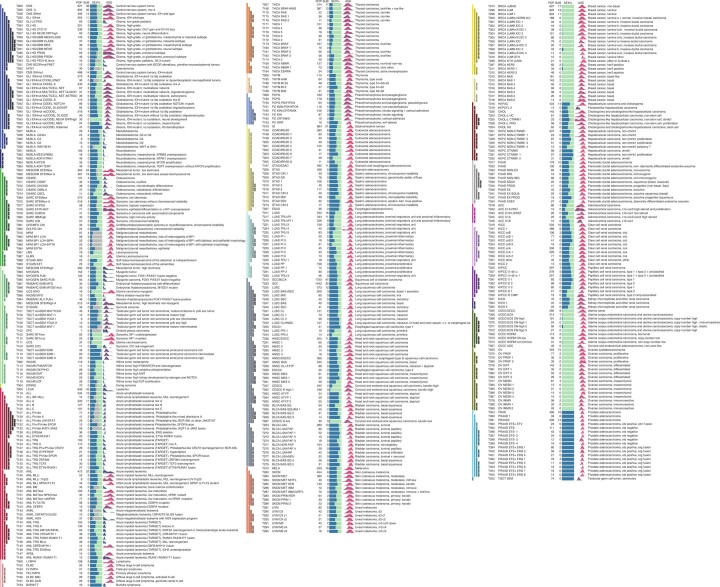
Pan-cancer hierarchy. Hierarchical list of the clusters obtained with RACCOON on the reference tumor dataset. For each class, sex ratio, age distribution, code, population, and short name are shown. These clusters have been then used as target classes for the CNN classifier.

**Extended Data Fig. 3:**
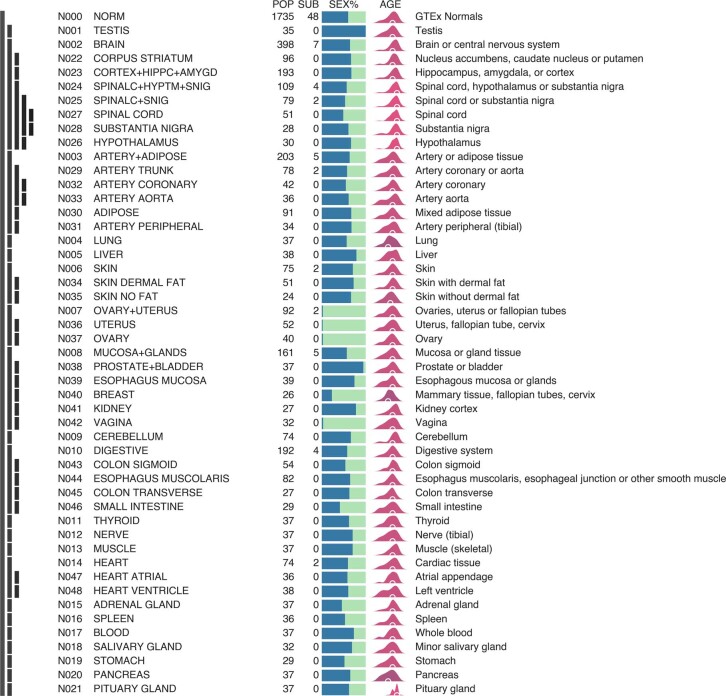
Hierarchy of all normal tissue classes. Hierarchical list of the clusters obtained with RACCOON on the reference normal dataset. For each class, sex ratio, age distribution, code, population, and short name are shown. These clusters have been then used as target classes for the CNN classifier.

**Extended Data Fig. 4:**
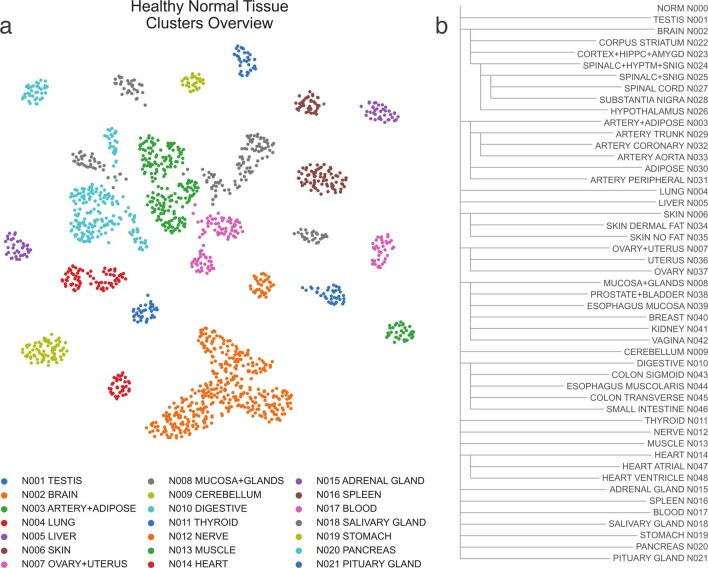
UMAP projection of normal tissue samples. (**a**) 2d UMAP representation of the hierarchical branch of healthy normal tissue samples included in our study. Different families are shown with different colors. (**b**) Dendrogram representing the same hierarchy, showing the connection among different normal tissue subtypes. As for the neoplastic hierarchy, samples are first grouped by tissue with exceptions. For example, breast tissue samples with a majority population of adipose cells by histology are grouped with other adipose-rich samples (N030), while those with most mammary gland tissue are found with other glands (N008).

The 13,313 tumor and non-neoplastic samples were divided into 455 classes, arranged across eight levels (Fig. 2a,b, Extended Data Figs. 2–4, Supplementary Fig. 7 and Supplementary Tables 1 and 2). There were 26 main tumor types at the top-most level, which were further divided into up to 48 subtypes each. To better understand the structure of these trees, we developed a score to measure the relative size of offspring branching along the hierarchy tree, called the Population-Weighted Splits (PaWS) (Fig. 2b, Supplementary Fig. 8 and Methods). Four main tumor types with the highest PaWS scores (deepest branching) encompassed a large proportion of the entire cohort. Together, the pan-leukemia group (T005 LEUK), squamous cell cancers (T012 SCC/BLCA), central nervous system tumors (T000 CNS) and sarcomas (T002 MESODM STEMlow and T003 MESODM STEMhigh) accounted for nearly 39% of all tumor samples. In total, 192 tumor subtypes descend from these five tumor clusters.

Tumors from a similar tissue typically co-clustered at the top level, as expected^22^. New factors emerged as drivers of transcriptional difference when looking within trees and exploring their structure. Age is an important factor—the distribution of cancer types differed between adult and childhood cancer. Eighty-five percent of pediatric cancers belonged to only six of 26 top-level types, but these were more likely to involve deep subtypes (mean PaWS, 0.83 versus 0.50; mean number of offspring, 22.6 and 12.2), many of which represented novel cancer subtypes.

Similarly, non-neoplastic samples were first grouped by tissue of origin, yet, occasionally, the transcriptional stratification transcended the organ of origin (Extended Data Figs. 3 and 4).

To further define the transcriptional subtypes of childhood cancer, we performed an in-depth annotation of 162 clusters representing the major pediatric tumor families. We noted their changes in survival, age, sex and underlying genomic alterations where possible, as well as key genes differentiating them from their adult counterparts. The clusters detailed in this manuscript did not have statistically significant differences in sex ratio.

### Intrinsic disorder of childhood tumors

Having defined childhood-specific cancer subtypes, we investigated their internal differences in gene expression. We measured expression fluctuations at the level of individual genes across tumors whose overall transcriptional profiles were similar (that is, expression changes of the *same* genes *among* tumors in the same cluster). These fluctuations were quantified using Shannon entropy (*S*; Methods)^23^ that, in our context, can be thought of as the ‘transcriptional disorder’ of tumor subtypes.

Non-neoplastic tissue was less disordered than cancers. Normal cells appear to allow for a narrow range of expression, whereas tumor types can tolerate more variation in gene expression while still maintaining a characteristic expression profile (11% higher entropy on average at the first level; Supplementary Fig. 9). The same was true when comparing tumor clusters to their matching non-neoplastic types (average 10% higher *S*; Supplementary Fig. 10). Not only did tumor subtypes have a significant increase in transcriptional disorder compared to their normal equivalent, but there was a positive correlation between most (Pearson 0.56, *P* = 2.29 × 10^−2^). This suggests that a tumor’s transcriptional variability may be predetermined by its tissue of origin.

**Fig. 3:**
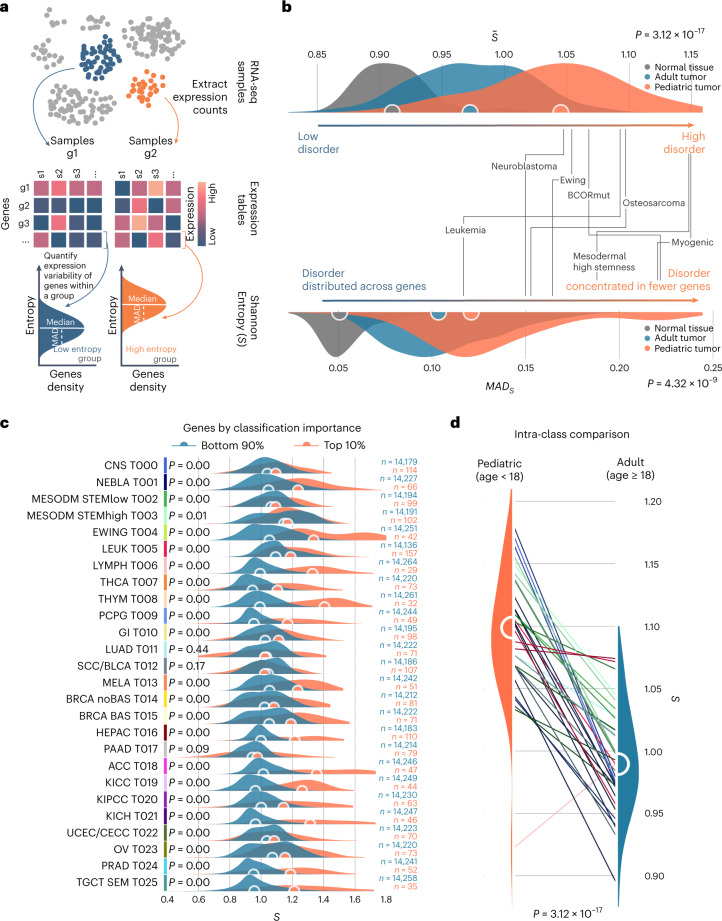
Expression entropy in childhood cancer. **a**, Schematic representation of the steps involved in measuring transcriptional entropy. Given groups (g) of samples and their expression tables, entropy is first measured for each gene within each group. Entropies from all genes are then pooled to define a distribution. When needed, the median entropy across all genes is used as a single score. **b**, Median entropy (*S*, top) and MAD (bottom) distributions observed in healthy normal (in gray), adult tumor (in blue) and pediatric tumor (in orange) subtypes as identified by our multiscale clustering algorithm. Median values are reported as circles. The two-sided Mann–Whitney *U*-test *P* values between these distributions are also reported; pediatric tumors are overentropic (*P* = 3.12 × 10^−17^) and have significantly higher MAD (*P* = 4.32 × 10^−9^) than adult tumors. Labels point at the values for selected tumor classes of interest. **c**, Per-gene entropy distributions for each of the 26 main tumor classes. The genes are grouped by their DeepLIFT importance score, where the genes summing to the top 10% cumulative importance are shown in orange, and the rest are shown in blue. The separation between these distributions is significant (two-sided Mann–Whitney *U*-test *P* < 0.05) in all cases, except for lung, pancreas and squamous cell. **d**, Intra-class entropy differences between pediatric (left) and adult (right) samples, for a selected group of classes populated by samples with a mixed distribution of ages. The pediatric samples show significantly higher entropy across all cancer types (two-sided Mann–Whitney *U*-test *P* = 3.12 × 10^−17^), leukemias (*P* = 1.79 × 10^−6^) and sarcomas (*P* = 2.71 × 10^−4^). Significance is maintained after the removal of classes outside the population interquartile (*P* = 1.18 × 10^−5^).

Childhood cancers typically have lower somatic mutation burdens^15^. As there are fewer mutations potentiating expression changes, one might expect a less noisy transcriptome. However, when looking within well-circumscribed tumor classes we found significantly higher transcriptional disorder in childhood cancer (Fig. 3b and Supplementary Table 1) across all cancer types. This holds true even after removing sampling bias by maintaining only classes within the interquartile. In all but two tumor types, cancers from younger patients had higher disorder than their adult equivalent (Fig. 3d and Supplementary Table 3).

We wondered whether the excessive transcriptional disorder seen in childhood cancer involved a subset of expressed genes or large parts of the transcriptome. This can be quantified by the median absolute deviation (MAD) of the per-gene entropy distributions: small values mean that most genes are similarly entropic, and high values mean that their disorder level can vary widely.

In childhood cancer types, the transcriptional disorder is broader, impacting different genes to different degrees, with a higher MAD score than adult tumors (Supplementary Table 1).

**Extended Data Fig. 5:**
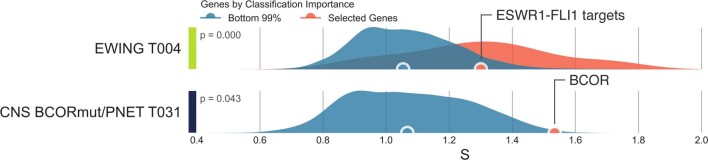
Entropy distribution Ewing and BCOR-rearranged sarcoma. Comparison of gene entropy distributions for Ewing (top) and BCOR-rearranged (bottom) sarcoma clusters. genes summing to the bottom 99% of cumulative DeepLIFT importance score are shown in blue, while in orange are a selected subset of tumor-defining genes (EWSR1-FL1 targets as defined by Zhang et al. Cancer Res. 2005, and BCOR respectively). *P* values from the two-tailed Mann–Whitney *U*-test are shown.

The most disordered genes represent marker lesions localized to a small portion of the genome and are remarkably specific to each subtype. We ranked the genes in input to our ensemble CNN by their relevance in identifying each tumor type with feature importance extraction (Deep Learning Important FeaTures (DeepLIFT)^24^). In most types, the top 10% cumulative importance genes are also those with the highest entropy (Fig. 3c). These correspond to disease-defining pathways (Extended Data Fig. 5 and Supplementary Table 4). Compared to adult malignancies, childhood cancers are mostly transcriptionally distinct, forming unique subtypes. However, within these subtypes of childhood cancer, there is remarkable flexibility among disease-defining genes.

### A stemness superclass of sarcoma

**Fig. 4:**
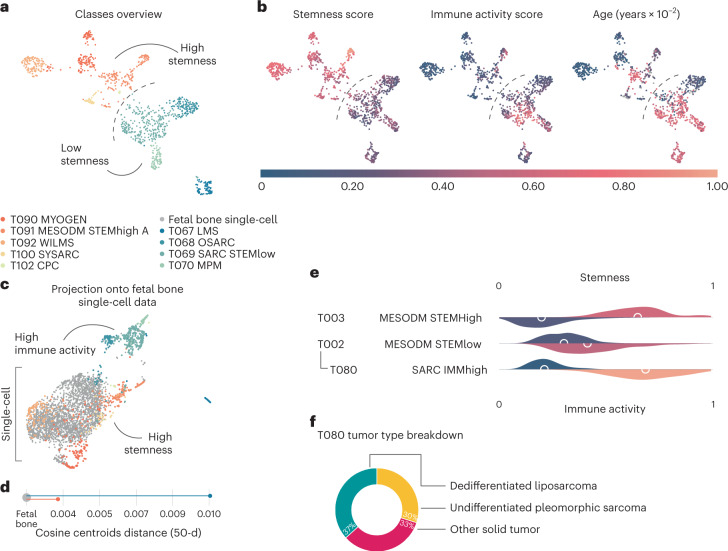
Clusters of mesodermal tumors. **a**, Two-dimensional UMAP projection of the mesodermal tumor classes by gene expression, highlighting the separation between high stemness (T003, in shades of orange) and low stemness (T002, in shades of green and blue) classes. **b**, The same map with samples colored by stemness score (left), immune activity score (center) and age (right). **c**, The same samples projected onto a set of single-cell datapoints from fetal bone tissue, showing how stem-high samples (T003) are more closely related to the embryonal tissue. **d**, Cosine distance of the centroids of mesodermal clusters from the fetal bone tissue obtained from a 50-dimensional (50-d) UMAP projection. **e**, Normalized stemness (top of each class, two-sided Mann–Whitney *U*-test *P* = 4.65 × 10^−165^) and immune activity (bottom of each class, two-sided Mann–Whitney *U*-test *P* = 3.28 × 10^−102^) score distributions for a subset of mesodermal tumor classes. **f**, Composition of T080, a class of sarcomas with high expression of immune markers, by diagnosis.

Sarcomas are proportionately more common in childhood. We identified 55 sarcoma and mesodermal solid tumor clusters, including 37 subtypes, most of which either contain a known fusion or are derived from a common tissue. One can clearly distinguish osteosarcoma (T068), leiomyosarcoma (T067), fusion-positive and fusion-negative rhabdomyosarcomas (T094 and T093), synovial sarcomas (T100) and others. Importantly, other cancers that are thought to derive from the mesoderm, such as mesothelioma^25^ (T070), Wilms tumor^26,27^ (T092), choroid plexus carcinoma^28^ (T102) and testicular non-seminoma germ cell^29^ (T101 and T105), also clustered with sarcomas, whereas Ewing sarcoma (ES) (T005) did not. Overall, the transcriptional contribution from the tissue of origin appears to be greater in sarcoma than carcinoma (Figs. 2a and 4).

ES is an example of the uniqueness of pediatric cancers as identified by RACCOON. ES forms a unique, separate cluster not only from other sarcomas but from all other cancer type. ES is one of 26 top-level tumor types and one of only three to have no descendants. These unique transcriptional features can be used as a straightforward diagnostic test. We found that up to 12% (9/80) of ES tumors by standard pathology may be misdiagnosed *CIC*-driven or *BCOR*-driven sarcomas (Supplementary Table 5).

All non-ES sarcomas were constrained to two mesodermal superclasses. The first (T002) comprised osteosarcomas, leiomyosarcomas, mesotheliomas and a diverse collection of less differentiated soft-tissue sarcomas found predominantly in adults. The second (T003) assembled rhabdomyosarcomas, synovial sarcomas and other predominantly pediatric sarcomas and mesodermal tumors. T002 and T003 were then subtyped into 24 and 29 mesodermal subclasses, respectively (Supplementary Fig. 8).

**Extended Data Fig. 6:**
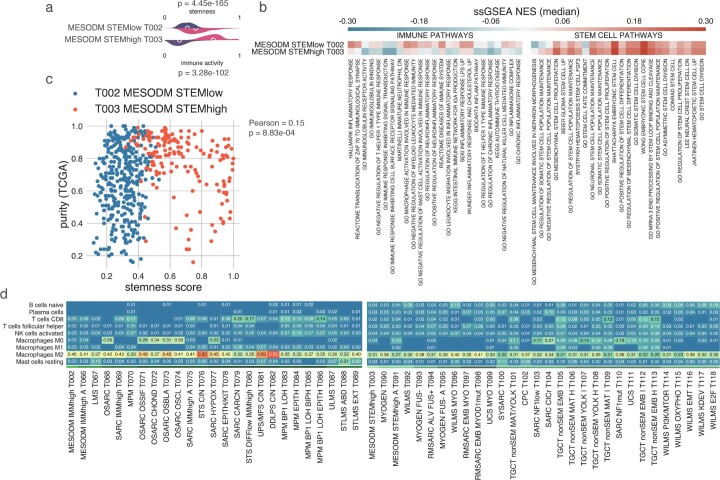
Comparison between high stemness and high immune activity mesodermal tumors. (**a**) stemness (top of each class) and immune activity (bottom of each class) scores distributions for the two main mesodermal tumor classes, T002 and T003. *P* values are from two-tailed Mann–Whitney *U*-tests. (**b**) Median normalized enrichment score for the top differentially regulated sets between T002 and T003 (GSEA one-sided hypergeometric test fdr < 0.001). (**c**) Tumor purity as a function of the stemness score across TCGA samples in T002 (in blue) and T003 (in orange). No correlation was found between the two values (Pearson’s correlation 0.15, two-tailed t-distribution p-val 8.64e-04). (**d**) Fractional immune cell type composition breakdown for tumor subtypes along the mesodermal hierarchy branches. These results were obtained with CIBERSORT and show a diversity of cell type abundances between the two groups.

Both mesodermal superclasses expressed epithelial-to-mesenchymal transition markers^30^ but otherwise had divergent expression programs. T002 sarcomas displayed high immune activation, with enrichment for pathways indicating a robust immune response (Extended Data Fig. 6b)^31,32^, more leukocytes^33^ and M2 macrophages (and M1 macrophages, to a lesser degree), along with high overall stromal content (Extended Data Fig. 6d).

For instance, T080 SARC IMMhigh is a small subclass of mixed solid tumors characterized by high leukocyte fraction, M2 macrophages and CD8^+^ cells. It is mostly composed of dedifferentiated liposarcomas (11/30) and undifferentiated pleomorphic sarcomas (9/30). Despite the variety of tumor subtypes, this class has a homogeneity of expression, possibly due to the immune transcriptional signal and the lack of idiosyncratic profiles because of their undifferentiation.

The second mesodermal superclass (T003) involved high markers of ‘stemness’. Stemness markers were among the most significantly enriched gene sets (*P* < 0.001; Extended Data Fig. 6b)^34^, confirmed using three independent methods^34–36^ (Methods). As others have noted^34^, we observed a negligible relationship between stemness and tumor purity (Extended Data Fig. 6c). T003 could represent a class of mesodermal cancers of embryonic origin. This notion is supported by the inclusion of rhabdomyosarcomas and germ cell tumors. To further explore this, we obtained tissue from a fetal sample estimated to be 56 days in postconceptual age, sequenced 37,490 cells and compared their expression profiles to that of the bulk-sequenced sarcomas. Overall, the T003 class of cancers was more similar to fetal cells, with some of its subtypes clustering immediately adjacent to in utero cells, supporting their early origins (Fig. 4c,d).

Taken together, these results support the idea that T002 (STEMlow) is a class of malignancies with more committed differentiation, characterized by high stromal content and an active immune profile. In contrast, T003 (STEMhigh) includes sarcomas with a more immature phenotype, possibly reflecting their embryonic origin. It is likely that their common mesodermal lineage brings these solid tumors together while keeping them apart from the rest of cancer.

### A diagnostic and prognostic aid for childhood cancer

RACCOON identified clusters for most major types of pediatric leukemia, brain tumors and solid cancers. For nearly every recognized pathological classification of pediatric cancer, there was a corresponding transcriptional cluster. For instance, in brain cancers, one can differentiate subtypes of medulloblastoma (T027), 1p/19q codel gliomas (T044), as well as those with/without *IDH1* mutations (T030 and T029), and ependymomas (T032), among others. Within the leukemias, one can differentiate *BCR*–*ABL1-*positive acute lymphocytic leukemia (ALL), as well as Ph-like variants (T127, T137, T139 and descendents) and distinct subclusters driven by fusions in *CBFB*–*MYH11* (T145), *PML*–*RARA* (T147), *TCF3*–*PBX1* (T135) and *RBM15*–*MKL1* (T515), as well as acute myeloid leukemia (AML) with *KMT2A* internal tandem duplications (T153), *KMT2A* rearrangements (T159) and additional leukemia subtypes (Supplementary Fig. 8). For rarer subtypes, we saw evidence for emerging clusters that may be further subtyped with the inclusion of more samples. Both established and novel subtypes of childhood cancer can be assessed using this transcriptome-based approach.

**Extended Data Fig. 7:**
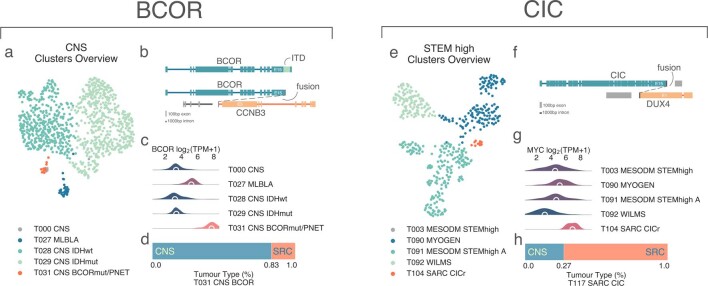
*BCOR*- and *CIC*-mutated tumors. Summary of the findings relating to *BCOR*-mutated and *CIC*-mutated tumors. (**a**) Two-dimensional UMAP projection of CNS tumors by gene expression, where a few representative classes are shown with shades of blue and green. The *BCOR*-mutated class is highlighted in orange (T031). (**b**) Diagram representing canonical *BCOR*-ITD and *BCOR*-*CCNB3* rearrangements. (**c**) *BCOR* expression distribution across representative CNS classes, showing a clear overexpression in *BCOR*-mutated samples (T031). (**d**) The idiosyncratic transcriptional profile of *BCOR* mutations is sufficient to overcome the cell-of-origin attraction during the clustering process. The ratio of tumor types within T031, shows that while it is mostly composed of CNS tumors, sarcomas are also found in this class. (**e**) Two-dimensional UMAP projection of MESODM STEMhigh tumors by gene expression, where a few representative subtypes are shown with shades of blue and green. The *CIC*-mutated class is highlighted in orange (T104). (**f**) Diagram representing the archetypical *CIC*-*DUX4* fusion. (**g**) MYC expression distribution across representative mesodermal classes, showing overexpression in T104. (**h**) As for *BCOR*-mutated samples, the ratio of tumor types within T104 shows a mixture of sarcomas and CNS tumors.

Different histotypes were occasionally brought together into one cluster, indicating unexpected, shared expression programs or a common cell of origin. Within the hierarchy of brain tumors was a small (*n* = 12) but highly specific cluster of young childhood tumors (average age, 4.5 years). This cluster (T031) was composed of both central nervous system (CNS) and extra-CNS cancers with *BCOR*-associated gene expression programs^37^ (Extended Data Fig. 7a–d). Validating this annotation, all but one sample contained *BCOR* alterations, including fusions, partial deletions and internal tandem duplications^9,38^. Similarly, the sarcoma branch contained a class of small round blue cell tumors of mixed origin that included both sarcomas and brain tumors, with an average age of 12 years (T117). All had expression patterns reflective of *CIC*–*DUX4* fusions (Extended Data Fig. 7e–h). Although these tumors can be difficult to diagnose^39^, our data support the notion that they are a distinct entity, independent of the location in which they arise^40,41^.

**Fig. 5:**
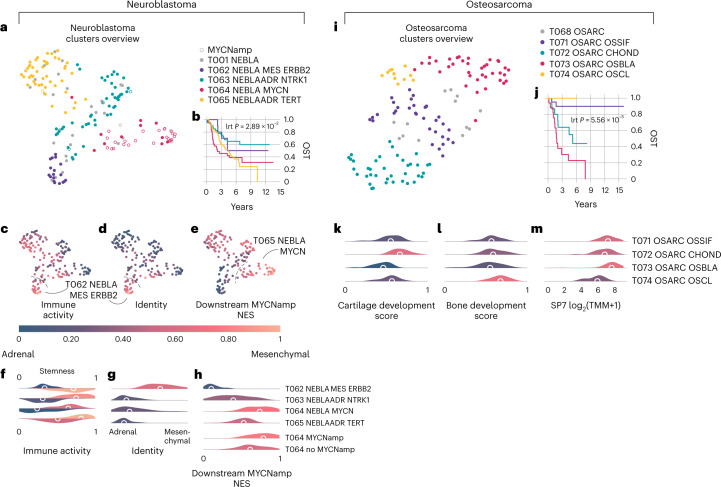
Subtyping of neuroblastoma and osteosarcoma. Summary of the findings relating to neuroblastoma and osteosarcoma tumors. **a**, Two-dimensional UMAP projection of neuroblastoma subtypes by gene expression. **b**, Overall survival curves for the neuroblastoma subtypes, showing significant prognostic stratification (log-rank test *P* = 2.89 × 10^−2^). **c**–**e**, The same projection but with samples colored as a function of immune infiltration (**c**), lineage (**d**) and normalized enrichment score (NES) of the expression pathway downstream to *MYCN* amplification (**e**). **f**, Distribution plots for the stemness (top of each class, Kruskal–Wallis test *P* = 8.10 × 10^−13^) and immune activity (bottom of each class, Kruskal–Wallis test *P* = 6.40 × 10^−11^) across neuroblastoma transcriptional subtypes. **g**, Cell lineage distributions grouped by neuroblastoma subtype (Kruskal–Wallis test *P* = 2.37 × 10^−2^). **h**, Normalized gene set enrichment score of genes expressed downstream to *MYCN* amplification, across neuroblastoma subtypes (top, GSEA one-sided hypergeometric test adjusted *P* = 2.26 × 10^−13^) and between samples in T064 clinically marked as *MYCN* amplified or not (GSEA adjusted *P* = 1.43 × 10^−4^). **i**, Two-dimensional UMAP projection of osteosarcoma subtypes. **j**, Overall survival time (OST) curves for the neuroblastoma subtypes, showing significant prognostic stratification (log-rank test (lrt); *P* = 5.56 × 10^−5^). **k**,**l**, Distributions of normalized gene set enrichment scores describing cartilage (**k**) and bone (**l**) development across the identified osteosarcoma subtypes. **m**, SP7 transcription factor expression distributions across osteosarcoma transcriptional subtypes.

**Extended Data Fig. 8:**
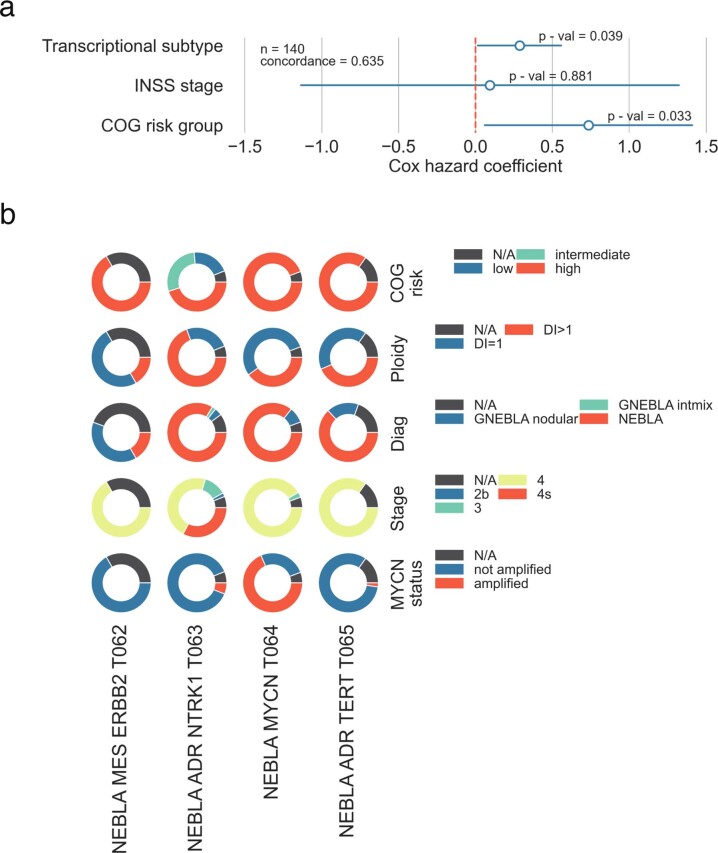
Neuroblastoma COX regression and clinical information. (**a**) Cox survival regression hazard coefficients calculated on neuroblastoma samples for three main covariates: the transcriptional neuroblastoma subtypes, INSS stage and COG risk group. Log-likelihood ratio test *P* values are shown for each covariate. Stratification by MYCN status was also included in the regression but failed the proportional hazard assumption test (*P* value = 6.8e-3). All covariates influence positively the hazard ratio, both the COG risk group and the transcriptional subtypes (T063, T062, T065, T064) terms are significant COG risk group has the most impact on survival with a coefficient of 0.73, while the transcriptional subtype hazard ratio is 0.29. Our stratification maintains significance in the hazard regression after accounting for the most powerful prognostic factors, suggesting it can capture otherwise unexplained contributions to the survival behavior and supporting its novelty in prognostics. (**b**) Clinical information from neuroblastoma patient data and its stratification by transcriptional subtypes. Shown are the proportion of patients with the following characteristics (from top to bottom), COG risk group, ploidy number, diagnosis (neuroblastoma or ganglioneuroma), stage and MYCN status.

Using the same approach, we found four subtypes of neuroblastoma, the most common childhood extracranial solid tumor (Fig. 5a). These subtypes, which overlap with previously reported clusters^42^, have substantial differences in immune activity, differentiation level and survival (Fig. 5b). Furthermore, their effect on survival is independent of Children’s Oncology Group (COG) risk group and stage (Extended Data Fig. 8). Named based on the expression of previously established marker genes *ERBB2* (T062), *NTRK1* (T063), *MYCN* (T064) and *TERT* (T065), these subtypes may be rooted in the tumor’s lineage^43–45^ (Fig. 5g). The *ERBB2*-overexpressing subtype is highly differentiated, with high immune activity, and reflected a neural crest cell/mesenchymal identity. Conversely, the *TERT* subtype is associated with a sympathoadrenal identity and has the highest level of stemness (Fig. 5g). These subtypes were only partially correlated with the established COG risk groups^46^, which are primarily based on histology and *MYCN* copy number. Of the 35 patients in the *MYCN* expression class, 25% (9/35) were not previously identified as *MYCN* amplified by standard testing but still maintained significant enrichment of downstream *MYCN* amplification pathways. That is, these patients with neuroblastoma had the transcriptional fingerprint of activated *MYCN* (Fig. 5h) yet would have been misclassified by conventional cytogenetics^47,48^.

Osteosarcoma, the most common bone tumor of childhood, was also readily subtyped using this method (Fig. 5i)^49^. We identified four osteosarcoma subtypes, separating by bone and cartilage development expression. The four subtypes also led to significant differences in prognosis (Fig. 5j). These include: a class characterized by osteoclast differentiation with good prognosis (T074); a second high-survival-rate class with enrichment of osteoblast differentiation and direct ossification (T071); a chondroblastic group with low to intermediate survival rate (T073); and a bona fide osteoblastic osteosarcoma class, with the poorest survival (T072). This demonstrates that whole-transcriptome profiling can unlock stratification with prognostic utility.

### Neural networks for diagnosing childhood tumors

**Fig. 6:**
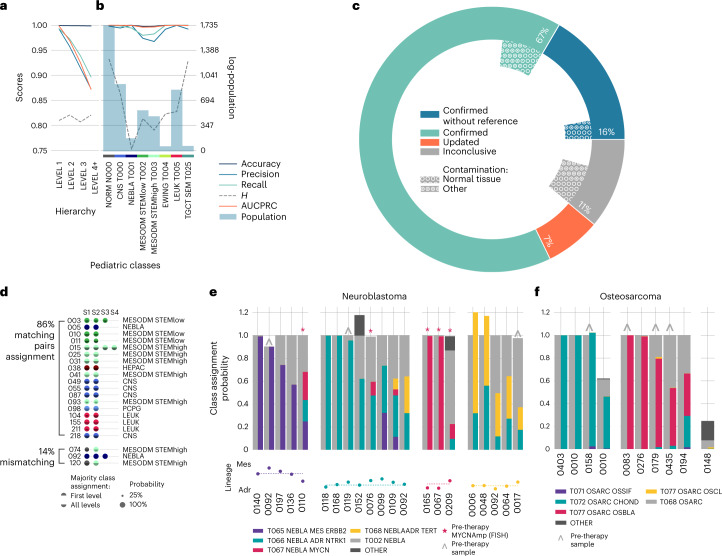
Diagnostic classification of childhood tumours. **a**,**b**, Classification scores obtained on the test set, broken down by hierarchy level (**a**) and by a subset of representative pediatric tumor classes (**b**). These include accuracy (dark blue), AUCPR (orange), precision (blue), recall (green) and hierarchical similarity *H* (dashed gray). All averaged scores were calculated as micro (m) averages. The total reference population of each class is also shown as shaded bars (blue). **c**, Classification results obtained with the KiCS validation dataset. In blue is the fraction of confirmed diagnoses in the absence of reference samples; in cyan are confirmed diagnoses; in orange are samples that led to an update in diagnosis; and in gray are inconclusive cases. The internal circle fractions indicate samples with normal tissue contamination (empty circles) or low quality (dotted circles). **d**, Majority class assignment for patients with samples taken at multiple timepoints. Each sample is shown as a dot, with size proportional to the class probability. The full circle represents the majority class at the first hierarchical level; bottom half circles in transparency show further subtypes. On the right, the name of the transcriptional family assigned to the first sample is shown in short form, except for those where normal contamination was dominant, in which case the next available sample is used. Samples with multiple separate primaries are not shown (Supplementary Fig. 11). **e**, Classification probabilities for neuroblastoma samples, grouped by their majority assignment. Larger bars represent the assignment to classes to the first level of the hierarchy; thinner bars represent the confidence scores of neuroblastoma subtypes. Samples for which *MYCN* amplification was clinically identified in a pre-therapy sample are marked with a red star. Pre-therapy samples are marked with a gray caret. The lineage score for each sample and their reference group median are shown at the bottom as dots and dashed line, respectively. **f**, Class assignment probabilities for osteosarcoma samples, grouped by their majority assignment. Larger bars represent the assignment to the osteosarcoma or alternative classes; thinner bars represent the confidence scores of osteosarcoma subtypes. Pre-therapy samples are marked with a gray caret.

Having determined that RACCOON can be used as a diagnostic and prognostic aid for childhood cancer, we validated an ensemble CNN (OTTER) to prospectively classify new patients’ tumors. OTTER outperformed current alternatives (Supplementary Table 6), reaching >0.99 mean area under the precision recall curve (AUCPR) across major pediatric malignancies while maintaining excellent performance even for minor subtypes deep in the hierarchy (Fig. 6a).

Tumor-derived RNA from childhood cancer patients enrolled in an ongoing precision medicine program were sequenced (163 tumors/132 patients)^50^. OTTER was applied to this held-out validation cohort, and classifications were compared to the pathologists’ diagnosis (Supplementary Table 7a). These patients are representative of the hard-to-cure tumors seen at most large childhood cancer centers: 44% (72/163) were relapsed, refractory or metastatic disease; and 60% (97/163) were obtained after one or more therapies.

OTTER’s tumor classification was concordant with the pathologists’ diagnosis for 65.6% of the cases (Fig. 6b). In an additional 16.6% of cases, we confirmed the diagnosis even in the absence of a corresponding tumor type in our reference set by comparing their class assignment to similarly labeled samples in both the reference and the validation cohort (Methods). Of note, the diagnosis was updated for 11 cases from nine patients (additional 6.7% of cohort), including four *BCOR*-rearranged sarcomas, a kidney clear cell sarcoma with a *BCOR* internal tandem duplication (ITD)^51^, two infant lymphoblastic leukemias with *MLL* partial tandem duplications and two megakaryoblastic leukemias with sarcomatous components. Altogether, OTTER’s classification was correct, in that it either matched or refined the pathologists’ diagnosis, for 88.9% of cases.

Because OTTER’s prediction probabilities are multiclass (samples can be assigned to more than one class), we could identify samples with high contamination by non-tumor tissue. Normal tissue expression was the dominant profile in 4% (6/163) of the samples and present to a lesser degree in an additional 4% (7/163) of tumors. Overall, there was no correlation between tumor cellularity and the confidence to which the tool assigned each specimen. Indeed, we confirmed the diagnosis of 89% (16/18) of the samples with <50% tumor purity. Nine percent (15/163) of the entire cohort showed signs of necrosis or other quality-related issues. Six samples (4%) remained inconclusive due to the current lack of a comparable match in the reference hierarchy.

To evaluate the robustness of OTTER’s predictions over time, we sequenced multi-timepoint samples (for example, primary metastasis pairs). Twenty-one patients had more than one tumor sequenced (Supplementary Fig. 11). Eighty-six percent of these cases maintained consistent class assignments over time (Fig. 5c), with the exception of two Wilms tumors with contamination as well a *SMARCB1*-associated tumor, a subtype currently absent from our reference. Taken together, OTTER’s tumor type predictions are highly concordant with those of pathology, can help to clarify ambiguous diagnoses and stay consistent across time even as the tumors evolve.

From this temporal analysis, the only tumor to markedly switch its transcriptional profile at relapse was a neuroblastoma (0092 in Fig. 6d). To explore this, we measured variability in class assignment of all neuroblastomas (including those with one timepoint). Individual neuroblastomas expressed multiple transcriptional programs at the same time (Fig. 6e). More than half of the available neuroblastoma samples (11/21) comprised more than one subtype (with >2% confidence). Neuroblastomas that had been clinically subtyped as *MYCN* amplified at diagnosis displayed a highly variable *MYCN* signature at relapse (subtype T077). The heterogeneous assignment of neuroblastoma subtypes seems to be unique among well-characterized tumors. In contrast, all but one of the sequenced osteosarcomas were assigned to a unique subtype (Fig. 6e). Neuroblastomas can maintain distinct states^43,44^. Our data indicate that neuroblastomas’ plasticity can be observed and quantified in vivo without single-cell analysis.

## Discussion

Pediatric cancers are the most common cause of death by disease among children in the developed world. Our data quantify their heterogeneity and provide a molecular definition for every major type of childhood cancer. Because these definitions are based on transcriptional profiles rather than mutations or methylation signatures^52^, they represent the active state of the disease. The recurring theme that emerges from this work is the transcriptional variability of childhood cancer. Childhood cancers are rooted in fewer major tumor classes—85% are in only six major classes—but then display deeper, more complex hierarchies. This suggests that many childhood cancer types share a common ancestry and then differentiate into a multitude of tumor subtypes.

Childhood tumors were less likely to fully match the stereotypic expression profile of their subtype. That is, there was greater transcriptional diversity among individual childhood tumors, even those belonging to the same subtype. Although bulk sequencing does not permit direct cell-to-cell comparisons, we can speculate that this diversity reflects heightened inter-cellular heterogeneity in pediatric cancer. Elevated transcriptional diversity may come from the embryonic stem cells from which some childhood tumors have been shown to be derived^53^. Like embryonic cells^54^, childhood cancers may use their ‘noisy’ expression to dynamically adapt their transcriptional programs.

Our assessment of childhood cancer transcription revealed other features that similarly pointed toward the developmental roots of many, if not all, pediatric tumors. Sarcomas are a broad class of tumors diagnosed disproportionately in the first three decades of life. They separated from all other cancers at the top-most level of our cancer hierarchy in two distinct groups. One of these (T003) was mostly made up of multiple childhood sarcomas, all segregating because of strong features of stemness and stem-like expression programs.

**Extended Data Fig. 9:**
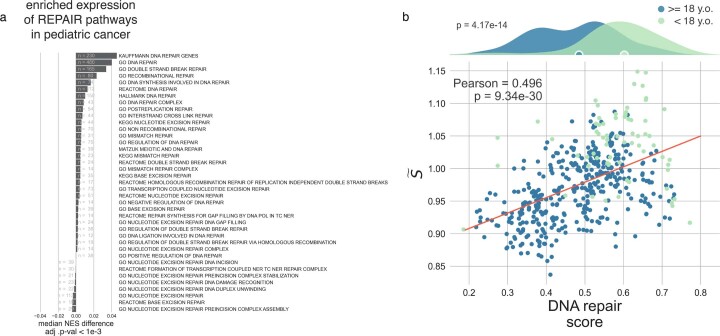
DNA repair pathways. (**a**) Significantly enriched DNA repair pathways in pediatric cancers when compared to adult malignancies. The normalized enrichment scores were obtained with TMM-normalized expression pre-ranked GSEA. Only sets with one-sided hypergeometric test adjusted *P* value of less than 0.001 are shown. (**b**) Scatter plot showing values of median class entropy as a function of the DNA repair score (See Supplemental Methods for a definition). Adult classes are shown in blue, pediatric classes are shown in cyan. In red is the result of a linear regression, Pearson’s correlation coefficients and one-sided *t*-test *P* value are reported. The distributions of the DNA repair score for adult and pediatric samples are shown at the top, together with the two-sided Mann–Whitney *U*-test *P* value giving significance to their separation.

The transcriptional variability of childhood cancers is in stark contrast to the quietness of their genomes, generally harboring fewer substitution mutations at diagnosis^55^. This low mutation burden is usually attributed to a limited number of cell divisions after fertilization and limited exposure to mutagens. Another possibility is that transcription itself facilitates or directs DNA repair. We observed that most DNA repair pathways are overexpressed in childhood tumors (Extended Data Fig. 9a and Supplementary Table 8); we also observed a significant correlation between transcriptional entropy and enrichment of DNA repair (Extended Data Fig. 9b). This includes overexpression of base excision repair pathways, which can regulate transcriptional fluctuations^56^, similar to what we observed in childhood tumors.

Having quantified their unique transcriptional features, we developed a diagnostic tool for childhood cancer. Using CNNs trained on 455 transcriptional classes, we matched or refined the pathologists’ diagnosis for 89% of patients. This tool is blinded to tumor site, morphology or immunophenotype and can accurately classify ~90% of childhood cancers using a small number of reads (Fig. 1b) and complements a DNA-methylation-based classifier for CNS tumors (Methods and Supplementary Table 7b)^52,57^. The tools described here also have prognostic utility, one example of which is in osteosarcoma where four subtypes with clear differences in survival were found. Instead of giving each tumor a single discrete label, our multiclass models can reveal expression of more than one subtype within a bulk tumor. This was the case for more than 50% of neuroblastomas, even switching dominant lineages after therapy. Our findings support current tumor-agnostic approaches, aiming to develop treatment strategies based on tumor biology^58^ rather than histology. These tools, and the taxonomy of cancer that underpins them, will continue to improve as more data accrue, yielding more accurate diagnoses and finer-grained subtype details—for every 10% increase in samples, up to an additional 10% of tumor subclusters are found (Supplementary Fig. 12). Thus, what is presented here is the first iteration of an ever-learning tool. Looking forward, our results indicate that this tool has the potential to grow such that it provides diagnostic or prognostic utility to every child with cancer.

## Methods

### KiCS enrollment and ethics declaration

The SickKids Cancer Sequencing (KiCS) Program is a prospective study of a demographically diverse population of children and adolescents and young adults (AYAs) with refractory, metastatic, relapsed or rare cancers, as well as children with unresolved suspicion of cancer predisposition. It was launched in April 2016 and is an ongoing study. Guardians or capable patients are guided through an informed consent discussion with a trained genetic counsellor or pediatric oncologist. KiCS has been approved by The Hospital for Sick Children’s Research Ethics Board. The first trimester human fetal tissue was collected from an elective termination of pregnancy procedure at Addenbrooke’s Hospital through the ethically approved Wellcome-MRC Cambridge Stem Cell Institute and Department of Clinical Neurosciences tissue bank (REC-96/085). Written informed consent was given for tissue collection by the patient in accordance with the Declaration of Helsinki 2000.

### Reference dataset

We used the UCSC Treehouse Childhood Cancer Initiative Compendium (version 9, March 2019)^9^ as a reference dataset to build the hierarchy of subtypes and train the ensemble CNN classifier. This cohort includes 11,750 tumor samples from TCGA^16^, TARGET^17^ and other contributing institutions, prepared with either poly(A) selection or ribosomal depletion. Gene expression counts from the STAR + RSEM Toil RNA-seq pipeline^59^ of samples in the compendium are publicly available and cover more than 58,000 genes, raw or normalized by log_2_ transcripts per million (TPM). The same pipeline was applied to any other data in this publication. Alternatively, counts obtained with Kallisto were used for performance benchmarks. To expand the pediatric reference, we added 313 further samples from St. Jude Children’s Hospital Pediatric Cancer Genome Project (PCGP)^18^, run through the same pipeline after filtering them by alignment quality. While building the subtypes hierarchy, we removed samples with particularly low purity but kept them to boost the ensemble CNN training. Ribodepleted samples showed consistent batch effects across tumor types during the clusters search. We, thus, chose to exclude them from the rest of the analysis and the CNN training; ribodepleted-only classes were removed from the hierarchy. Finally, we added 1,735 normal tissue samples with the best coverage and quality scores from 51 different organ sites from the GTEx project^19^ to the dataset. To avoid degradation in the output from tumor samples with normal contamination within the Treehouse cohort, the tumor and normal datasets were kept separate at the first stage of the clustering and merged later as separate branches of the classification hierarchy.

In input to the clustering algorithm, genes mapping to non-coding sections of the RNA were removed. Among these remaining, only genes with high variability, accounting for 99% of the cumulative variance on the full cohort, were kept. This reduced the feature space to 18,010 functional genes and pseudogenes, allowing us to speed up the rest of the analysis with a negligible loss of information.

Diagnoses and genomic markers reported by the sharing institutions were used, when available, as a reference for tumor type comparisons and the annotation of clusters.

### Quality control and batch effects

Samples included in the final version of our reference cohort were pre-filtered by standard quality control parameters by the groups that generated the data^9,18,19^. We included GTEx samples with a high number of sequenced reads (as a proxy for coverage). St. Jude samples from the PCGP cohort were filtered based on TPM distribution. Samples were ranked based on the number of protein-coding genes found to have zero expression (TPM = 0) and excluded if more than 25% of their protein-coding genes were not expressed. This resulted in an improvement in RACCOON’s clustering and clarity of each cluster (Supplementary Fig. 13). Any St. Jude sample already present in the Treehouse data was removed. This left us with 512 samples from the St. Jude cohort—62% of the total available.

### Differential expression and gene sets analysis

log_2_ TPM-normalized counts were used for clustering, classification and map projections. For differential expression analysis, TMM normalization and the negative binomial generalized log-linear model fitting from EdgeR^60^ version 3.30.3 were used instead. Gene set enrichment analysis (GSEA) and single-sample GSEA (ssGSEA) were carried out with gseapy^61^ version 0.9.5 in Python version 3.6.9 and GSVA^62^ version 1.36.3 in R version 4.0.2. ssGSEA-based scores were also calculated with gseapy version 0.9.5 on TPM-normalized counts and scaled between 0 and 1 to assure consistency in the comparisons. They were used as part of stemness, immune activity and neuroblastoma identity scores (see Stemness score, Immune deconvolution and activity score and Neuroblastoma cell lineage score in Methods for details). The two-sided Mann–Whitney *U*-test was used when evaluating significance in comparing these scores and any other distribution between paired groups of samples throughout the text.

Plots and diagrams were produced with Matplotlib^63^.

### Survival analysis

Survival analyses and log-rank tests were carried out with lifelines version 0.21 (ref. ^64^). Where available, outcomes were defined based on overall survival times provided by the sharing institution. A Cox survival regression of neuroblastoma subtypes was performed with the same library on 161 neuroblastoma observations, of which 81 were censored.

### Multilevel clustering

Given a set of data points, RACCOON removes low-information features, reduces their complexity with a non-linear dimensionality reduction algorithm and finally identifies clusters with a density-based approach. The search is continued depth-first for each of the clusters identified iteratively. The search is terminated only when further splits would lead to a particularly suboptimal value of the objective function or the class population is lower than a pre-set bound (for example, 25–50 samples). The features removal cutoff, the number of neighbors employed by uniform manifold approximation and projection (UMAP)^65^ and the clusters search parameter (for example, maximum clustering distance parameter in DBSCAN) are optimized by maximizing a clustering quality score. For this project, the tunable parameters were optimized with a grid search, and the total silhouette coefficient of the dataset^66^ was set as the objective function. This score quantifies the quality of clustering by calculating the ratio between the clusters cohesion and their separation. Ranging between −1, when all points are incorrectly assigned, and 1, when all points are well assigned, we set here 0 as the minimum threshold for accepting a set. In a scenario where the best combinations of parameters found still leads to a negative score, the cluster under scrutiny is not split.

We applied RACCOON to our extended dataset to build a hierarchical tree of tumor and normal subtype clusters. The number of final (reduced) dimensions was empirically set to 12, a choice that proved to be a good compromise between accuracy and computational cost. A population cutoff of 25 was applied to stop the search, and nodes with fewer than ten samples were pruned, because their annotation and training for the classifier would be too unreliable. This method initially yielded more than 700 individual clusters. A subset of low-population leaf nodes was removed after manual annotation, for the lack of sufficient biological and gene expression information to support any finding, together with classes populated exclusively by ribodepleted samples. This process left us with a total of 455 clusters (406 tumor and 49 normal tissue classes, respectively), of which 303 are non-overlapping independent terminal (leaf) nodes.

### Normal tissue inclusion

Multilevel clustering was applied independently to the normal tissue samples and malignant subsets. Normal and tumor samples from the same organ would have been grouped in common classes at the highest level if they had been mixed. Clustering quality decreases, as less aggressive or low-purity tumors can be difficult to separate from healthy normal samples.

During training, this choice forced OTTER to learn high-level features that distinguish normal tissue from neoplasms, independently from their anatomical location. This boosted OTTERʼs ability to recognize tumor populations in low-quality samples, as the tumor–normal separation is prioritized in the hierarchy. Similarly, we expect generalization to unseen normal tissues to also be improved.

### Annotation

Clusters obtained with RACCOON were annotated based on their most characteristic transcriptional features compared to the closest members of their hierarchical family. Differential gene expression and GSEA were carried out to identify each clusterʼs defining gene and pathway expression. Limited clinical information, including age, sex and a diagnostic label, were available for each sample. All 455 separate classes were first annotated to assign a unique label (a code and a name). We then extended the annotation for the five major families of pediatric tumors: CNS, leukemia, neuroblastoma and the two mesodermal classes, as well as the branch stemming from the healthy normal samples. More details on the annotation of these groups can be found at https://rna-atlas.github.io/.

### Entropy calculations

Expression variance and its derived quantities (for example, the coefficient of variation) could be used as a proxy of variability; however, they fail when dealing with multimodal or discontinuous distributions. Shannon entropy *S* is a much more appropriate and robust measure. It is a generalization to Boltzmannʼs thermodynamics entropy; it quantifies the information content or the randomness of a given distribution^23^. It is defined as follows:

$$S = - \mathop {\sum}\limits_i {P_ilogP_i}$$

where *P*_*i*_ is the probability of an event *i*, in our case the probability that a certain gene will lead to a specific expression count.

Starting from TMM-normalized data, which already account for the skew introduced by extreme values across the population, the expression was standardized along genes to limit heteroscedasticity. Being additive, Shannon entropy cannot be naively measured on groups with different populations, and it requires enough samples to approximate a continuous distribution. In our case, this holds true for a good number of classes but not for the smallest leaf nodes. We, thus, first approximate the expression distribution of every single gene independently with a fixed-bandwidth Gaussian kernel density estimation and then extract the probabilities for Shannon entropy from the estimated distribution on a 100,000-points grid. Higher-resolution grids approach the limit of differential entropy and approximate the integral better, yet they lead only to marginal changes and increase the computational cost considerably. A mesh to 250,000 points led to a change in entropy of less than 2%, confirming that our choice was close to convergence. The entropy calculation was limited to a subset of more than 14,000 highly variant genes, by filtering those with both consistent low expression across samples and low entropy across all classes. The final values obtained for each gene and each class were divided by the median entropy of the normal tissues cohort class N000. All calculations were carried out with scikit-learn 0.22.2.post1(ref. ^67^).

RNA-seq expression data are commonly approximated by a negative binomial distribution, which accounts for overdispersion in its mean–variance relationship. The coefficient of variation is a popular measure of mean independent dispersion; however, it still relies on variance and, thus, inherits all its shortcomings when attempting to quantify transcriptional noise.

We observed a fair correlation between entropy and mean expression across all groups (Pearson *r* = 0.68). We, thus, fit a linear model on the entropy and adjusted the score to account for its dependency on the median expression (Supplementary Fig. 14). The coefficient of determination R^2^ was 0.46, suggesting that the mean dependent component accounts for less than half of the total entropy, and transcriptional noise within and across tumor types cannot be entirely explained by differences in expression levels alone. This adjusted entropy (*S*) was used throughout the manuscript.

### PaWS calculations

Although entropy entails the overall variability of gene expression within a population, part of this can be translated into a different pattern of activation of relevant biological pathways, thus defining different tumor types. This inter-tumoral heterogeneity is explicitly accounted for by the cluster hierarchy itself. We can define a score based on the number of offspring nodes that a specific group generates and measure it on the classes that we identified. We call this PaWS and define it as follows:

$$PaWS\left( n \right) = \frac{{\left| {L_n} \right|}}{{\left| L \right|}}\frac{{{{{\mathrm{log}}}}\left( {\left| {root} \right|} \right)}}{{{{{\mathrm{log}}}}\left( {\left| n \right|} \right)}}$$

where $n = \left\{ {sample_1,\;sample_2, \ldots } \right\}$ is a set of samples identified as a cluster or node; *L* is the set of all leaf nodes *l*—that is, all the childless nodes, *L*_*n*_  is the set of leaf nodes that are offspring of *n*; and *root* is the hierarchical tree root, a set containing all our dataset samples. The PaWS of *n* is, thus, defined as the ratio between the cardinality (|*L*_*n*_,|) of *L*_*n*_ and the cardinality of L – that is, the number of leaf nodes that are offspring of *n*, over the total number of leaf nodes, weighted by the inverse of the log population ratio of *n*. This last term was added to account for the fact that smaller clusters will have less probability to be split by the algorithm.

### Correlation between heterogeneity scores

The relationship between these quantities is not trivial; we observed a weak correlation (Spearman rank test coefficient = 0.355, *P* = 1.120 × 10^−5^) between median entropy and PaWS score, after removing all the leaf nodes, to avoid including clusters with possible subtypes but insufficient population to be split by the algorithm. Entropy is thus a good proxy for intra-cluster expression disorder, as it accounts for that part of expression differences within a population that are not coherent enough to be translated into clear subtypes and yet not able to disrupt the overarching patterns that define the parent class.

### Stemness score

A unified stemness score was calculated as the average among CytoTRACE^35^ single-cell stemness score, mRNAsi^36^ and the ssGSEA score from Miranda et al.^34^. The score was then normalized for each inter-tumor type comparison.

### Immune deconvolution and activity score

The immune activity score was calculated as the average between Reactome immune system^31^ ssGSEA score and Gene Ontology immune activity^32^ score. The result was averaged with methylation-derived leukocyte content fraction by Thorsson et al. (218)^33^ in TCGA samples where the information was available. The score was then normalized for each inter-tumor type comparison. Immune deconvolution scores and immune cell type ratios were obtained with CIBERSORT^68^.

### Neuroblastoma cell lineage score

A unified neuroblastoma cell lineage score was calculated by first averaging separately neural crest-like and mesenchymal identity ssGSEA scores and adrenergic identity scores from three different publications^43–45^. For each sample, the final score was obtained as the difference between the mesenchymal/NCC-like unified score and the adrenergic unified score, and it was scaled to range between 0 (more adrenergic) and 1 (more mesenchymal).

### Single-cell RNA-seq

Fetal age (post-conception weeks (PCWs)) was estimated using the independent measurement of the crown rump length (CRL), using the formula PCW (days) = 0.9022 × CRL (mm) + 27.372.

Paired femora, tibiae and fibulae were dissected from the fetal hind limbs by a specialist bone and soft tissue pathologist (P.B.) under a microscope using sterile microsurgical instruments. The femora were further dissected into proximal and distal halves, to give eight samples in total (paired proximal and distal femora, paired tibiae and paired fibulae). Each sample was then processed into single-cell suspensions. In brief, tissue was digested in a 5 µg ml^−1^ Liberase TH working solution prepared from Liberase TH powder (Sigma-Aldrich, 5401135001) and 1× PBS on a shaking platform (750 r.p.m.) at 37 °C for 30 minutes. The tissue was gently agitated using a P1000 pipette after 15 minutes. Then, 5 ml of 2% FBS in PBS was added to stop the dissociation, before second-stage digestion with 0.25% trypsin solution for a further 30 minutes at 37 °C, with pipette agitation every 5 minutes. Cells were then spun down at 750*g* at 4 °C for 5 minutes and resuspended in 50–200 µl of 2% FBS in PBS. Fetal cells were loaded for single-cell RNA-seq directly after sample processing.

Single-cell suspensions from the eight samples were loaded onto a separate channel of a Chromium 10x Genomics Single Cell 3′ v2 library chip as per the manufacturerʼs protocol (PN-120233), aiming for a cell capture recovery of 3,000–5,000 cells. cDNA sequencing libraries were prepared according to the manufacturerʼs protocol and sequenced on an Illumina HiSeq 4000 (2 × 50-bp paired-end reads).

Raw sequence reads in FASTQ format from fetal samples were processed and aligned to the GRCh38-1.2.0 human reference transcriptome using the Cell Ranger version 2.1.1 pipeline^69^ (10x Genomics) with default parameters.

The resulting expression matrices were processed with SoupX version 1.3.0 (ref. ^70^) to estimate and remove cell-free mRNA contamination before analysis. Cells with fewer than 300 genes and more than 7,500 genes were filtered, as well as those in which mitochondrial genes represented 10% or more of total gene expression. A quantitative estimation of cell cycle stage was performed on the remaining cells with Seurat version 3.0 (ref. ^71^). Log-normalization was then performed before data scaling, which used cell cycle score, mitochondrial gene expression level and the total unique molecular identifiers (UMIs) per cell as regression variables.

We normalized the raw expression data to log_2_ (TPM + 1) and randomly selected 25,000 samples. The resulting dataset was merged with the bulk RNA-seq sarcoma data (T002 MESODM IMMhigh and T003 MESODM STEMhigh). A low-information filtering step was applied, to boost the signal-to-noise ratio and partially remove batch effects, before projecting the data to a lower-dimensionality space with UMAP^65^. The nearest neighbors cutoff was set as the square root of the total population. The centroid distance between T002 and single-cell data was constantly higher than that between T003 and the single-cell cluster, independently of how the dimensionality reduction was parametrized over a grid of combinations (Supplementary Fig. 15).

### Classification

We built a set of mono-dimensional CNNs, called OTTER, which takes the RSEM gene expression reduced output (18,010 log_2_ TPM genes) as input and returns the membership probability to any or multiple of the 455 hierarchical classes.

We trained these networks on the full reference cohort of more than 13,000 samples, which includes samples at a range of sequencing depths—a computationally expensive task. The resulting model proved markedly more accurate and robust than alternative classification methods, such as k-nearest neighbors, which are affected by tears, deformations and the partial loss of meaningful distances in the dimensionally reduced space, and have limited flexibility when dealing with multiple tumor components.

To identify optimal architectures, we emplyed Hyperopt, a Bayesian hyperparameter optimization library based on a Tree-structured Parzen Estimator (TPE)^72^. The micro-F1 (μF1) score was chosen as the objective function to guide the search. We enriched this group of models with a number of manually tuned architectures.

All models included one-dimensional convolutional (CV) layers followed by fully connected (FC) layers. The number of filters of CV layers was tuneable and shared across layers, and so was the kernel size. Each CV layer was followed by batch normalization and max pooling with fixed size 4 and stride 2. The size of hidden dense layers was also tuneable and halved at every successive layer, and dropout was activated at parametrizable percentage. The loss function was binary cross-entropy. Adadelta was set as optimizer with a starting learning rate of 0.001 and early stopping.

The top-scoring models among the pool of all candidates were subject to five randomized rounds of five-fold cross. The splitting into train and test sets was stratified to assure a proportional coverage for every class, and early stopping after three epochs was activated to avoid overfitting. Five candidate models were then selected according to their different performances on a set of scores including macro-F1 (MF1) and macro precision recall area under the curve (MAUCPR).

The final classifier was built as an ensemble average of a subset of these models, an unweighted arithmetic mean of three. The ensemble classifier led to an improvement in most scores while limiting the shortcomings of each single model and adding robustness to the final predictions. Finally, the models were trained on all available samples, with an adequate number of epochs to avoid overfitting for each separate case. A comparison with alternative tumor type classification RNA-seq models available in literature can be found in Supplementary Table 6.

The models were built as multilabel and multiclass; both the input labels and the output membership assignment are not exclusive to a single class. A post-processing step ensures consistency among the probabilities of classes within a family: if the classifier assigns higher probability to an offspring node than to its parent, the average of the two is assigned to both classes, and sibling nodes are adjusted accordingly. This correction is then propagated upward along the hierarchy.

The scores in output from the final ensemble are not binarized to allow the user a full picture of confidence scores. To make up for the strong class imbalance in the training dataset, we recalibrated these output probabilities.

We identified a binary classification cutoff that maximizes an adapted Youden *J* statistic (precision + recall) and then transformed the output scores linearly so that this cutoff value falls at 0.5 of the final probability. Although this change does not affect markedly the resulting output (.998 cosine similarity, .957 hierarchical similarity on the validation cohort), it helps relieve some of the overfitting on minority classes. The median cutoff was .585 (.651 for highest level classes, .425 for the lowest level, .543 for leaf nodes), suggesting that this is an overall balanced classifier.

The input data features were ordered by correlation following a quick agglomerative clustering on their log_2_-normalized expressions. The input gene arrays are scaled to a 0–1 range, and the labels were transformed to a one-hot Boolean encoding.

All models were built with Keras version 2.2.2 and TensorFlow^73^ version 1.10.1 backend. All code was run with Python version 3.6.9, and model training was run on our local high-performance computing machine with eight Xeon E5-2670 v2 @ 2.50-GHz or Xeon Gold 6140 CPU @ 2.30-GHz cores and 64 GB of RAM.

### Comparison to DNA methylation-based classifier

We compared the results of our transcriptional classifier to a DNA methylation-based classifier^52^ for a set of CNS tumors. In a previous work^57^, we profiled 252 high-risk pediatric cancers through multiple sequencing technologies. Sixty-three of these are CNS tumors with data from both DNA methylation and RNA-seq and can be directly compared.

After a manual curation, the methylation classifier matched these tumors to their presenting clinical diagnosis in 86% of the cases. The remaining 14% are either matched to a wrong subtype but within the correct parent family or do not match the expected subtype. The two classifiers agree in almost all of these cases, within the limits of tumor types available in the respective reference datasets, and complement each other in the few exceptions.

The dataset includes a number of tumor subtypes that are rare or absent in our reference cohort (atypical teratoid rhabdoid tumor, diffuse midline gliomas (DMGs) and meningiomas); for the purpose of this comparison, consistency in their assignment to a transcriptionally similar subtype (for example, all DMGs that were assigned to the same proximal subtypes of high-grade gliomas) was considered a match.

Among the 8% of samples matched only to the parent family according to DNA methylation, OTTER, our transcription-based classifier, could correctly identify the subtype of three samples: a medulloblastoma, called as retinoblastoma by DNA methylation; a low-grade glioma, instead of a high-grade glioma; and an ependymoma, whose methylation profile was reflecting immune infiltration. In two cases, the classifiers were in agreement, in spite of a mismatch with respect to the pathologist diagnosis: an IDH wild-type glioma and a medulloblastoma of the G3 subtype. Finally, there are three cases in which the transcriptional classifier fell short, where the DNA methylation matched the correct subtype: an ependymoma, which was not recognized by OTTER due to low purity and high immune infiltration, and two atypical teratoid rhabdoid tumors, a subtype that is absent in our RNA-seq reference.

Both classifiers can provide highly accurate predictions and complement each other in the most complex cases.

### Hierarchical similarity score

To evaluate the accuracy of predictions within the hierarchical framework, we employ the hierarchical similarity score (*H*), a union/intersection score based on the graph information content similarity (SimGIC) that measures the proximity of two points along the class tree while accounting for its structure and populations:

$$\begin{array}{ll}{\rm H}\left( {v_1,v_2} \right) = 1 - \Delta _{u/i}\left( {v_1,v_2} \right)\\ \qquad\qquad \, = \frac{{\mathop {\sum}\nolimits_{n \in nodes\left( {v_1} \right) \cap nodes\left( {v_2} \right)} {w\left( n \right)} }}{{\mathop {\sum}\nolimits_{n \in nodes\left( {v_1} \right) \cup nodes\left( {v_2} \right)} {w\left( n \right)} }}\\ \qquad\qquad \, = \frac{{\mathop {\sum}\nolimits_{n \in nodes\left( {{{\overrightarrow 1 }} } \right)} {w\left( n \right)\min \left( {v_1\left( n \right),v_2\left( n \right)} \right)} }}{{\mathop {\sum}\nolimits_{n \in nodes\left( {{{\overrightarrow 1 }} } \right)} {w\left( n \right)\max \left( {v_1\left( n \right),v_2\left( n \right)} \right)} }}\end{array}$$

where *v*_1_, *v*_2_ are the membership assignment probability vectors of two samples; *nodes*(*v*_*x*_) is the list of nodes or classes activated in such vectors; $nodes\left( {\overrightarrow 1 } \right)$ is the list of all nodes; and *w*(*n*) are the nodes’ weights. These are calculated as information content—that is, the probability of a sample falling into the lower node connected to the edge, which can be approximated to the class frequency of observations in the training dataset:

$$w_{SimGIC}\left( n \right) = - logp\left( n \right)$$

We also define the partial hierarchical similarity score (*η*), which looks only at the branches active in the ground truth while disregarding false positives:

$$\begin{array}{ll}\eta \left( {v_1,v_2} \right) = 1 - \delta _{u/i}\left( {v_1,v_2} \right)\\ \qquad \qquad = \frac{{\mathop {\sum }\nolimits_{n \in nodes\left( {v_1} \right) \cap nodes\left( {v_2} \right)} w\left( n \right)}}{{\mathop {\sum }\nolimits_{n \in nodes\left( {v_1} \right)} w\left( n \right)}} = \frac{{\mathop {\sum }\nolimits_{n \in nodes({\vec {1}})} w\left( n \right)\;{{{\mathrm{min}}}}\left( {v_1\left( n \right),v_2\left( n \right)} \right)}}{{\mathop {\sum }\nolimits_{n \in nodes({\vec {1}})} w\left( n \right)\;v_1\left( n \right)}}\end{array}$$

### Sequencing depth benchmarks

Stochastic subsampling of the total number of reads was repeated at set intervals for five chosen samples from the KiCS cohort—five times with different random seeds for each set threshold. Each original sample had at least 10^8^ reads (on paired FASTQ files), and its OTTER output was set as ground truth. Accurate classification (.85 with RSEM, .75 *H* with Kallisto) can be obtained with OTTER with 1 million reads. Although less accurate, Kallisto is considerably faster (Supplementary Fig. 6).

### Library preparation and storage benchmarks

OTTER was trained on a dataset of poly(A) sequencing samples from fresh-frozen (FF) tissue. To evaluate its generalizability to alternative library preparation techniques, we tested its performance on a set of 247 samples from the Treehouse Childhood Cancer Initiative compendium version 9 (ref. ^9^) treated with ribosomal depletion (Supplementary Fig. 16). The closest possible tumor class to the provided diagnostic label was set as ground truth. Tumor subtypes that were absent in our reference were removed from this analysis; however, tumors lacking a matching class in the atlas, but with a consistent population in the reference cohort, were included. As an example, gastrointestinal stromal tumors, which are consistently found in the T078 SARC EPITH/KIT class but lack for now their own subgroup due to a limited population (*n* = 6), were included, and so were samples with myofibromatosis. The classifier can still identify the correct tumor type, albeit with lower confidence. Although ribdopleted libraries are somewhat compatible with our classifier, the results should be treated cautiously, and the user should be aware that different tumor types will have an unequal impact on the classifierʼs performance.

Formalin-fixed, paraffin-embedded (FFPE) is commonly used for long-term storage of samples, yet degradation of the DNA and RNA in FFPE samples has been described in literature, and most molecular-based analyses seem incompatible with FFPE data^74,75^ Information on the storage method is available for only 93 of the 247 samples in the Treehouse cohort. We then repeated the analysis on a smaller cohort of matched FF and FFPE samples (*n* = 52 pairs). This set includes a slice of the KiCS cohort and samples from two different publications^75,76^. We also stratified the FFPE tumors by library preparation to demonstrate that the impact of library preparation and storage are additive (Supplementary Fig. 16).

### Expanding the tumor atlas

We investigated the behavior of OTTER in inference on data from tumor types missing from our transcriptional phenotypesʼ hierarchy, and we measured the effect of adding such data to the RACCOON clustering. To this end, we selected 19 atypical teratoid/rhabdoid tumors (AT/RTs) from an unseen dataset, which was not used for clustering or training, and ran them through the current version of OTTER. Eighteen AT/RT snap-frozen tumor materials and clinical information were collected at The Hospital for Sick Children or through an international collaborative network with consent as per protocols approved by the hospital research ethics boards at participating institutions. All AT/RTs had negative BAF47 immunohistochemistry stain and biallelic *SMARCB1-*inactivating alterations as confirmed with FISH, MLPA, targeted Sanger sequencing or high-throughput sequencing analyses. RNA-seq libraries were prepared using the Illumina TruSeq RNA Sample Preparation Kit for poly-adenylated mRNA selection and sequenced at the Centre for Applied Genomics^77^. To these, we added a single AT/RT sample from the KiCS cohort.

Our reference cohort currently contains three samples that have been labeled as AT/RT. Two of these are found in T040 GLI HG/GBM MES, a group of high-grade gliomas and glioblastomas of the mesenchymal subtypes. The remaining sample was grouped by RACCOON with lung adenocarcinoma, likely due to contamination or low tumor purity. A true AT/RT target class is not available to OTTER. Fifteen of 19 samples are assigned to classes along the T040 CNS branch with at least 5% of confidence. AT/RTs also possessed some signal of high stemness, yielding a partial match to the mesodermal stem high class (T004) in 15 of 19 samples.

We then clustered the group of 21 AT/RTs (19 unseen + 2 high-quality samples from the reference cohort) using RACCOON together with samples from the CNS class (Supplementary Fig. 17).

All AT/RTs clustered together within a new class (just below the high-grade gliomas T034, in the same lineage as T040). This demonstrates that a critical threshold of AT/RTs was reached to create a new subtype. To study what the exact threshold is, we performed subsetting experiments. Clustering was repeated using different numbers of AT/RT samples along with 100 other CNS tumors, both of which had been randomly selected five separate times. TPEs were used to speed up the search for repeated runs. We computed the adjusted mutual information (AMI) score on the clustering result by assuming a perfect separation of AT/RTs from all other CNS samples as ground truth, to assess how close the resulting partition was to having an AT/RT-only class (Supplementary Fig. 17).

### Population characteristics

We included several publicly available, uniformly processed cancer transcriptomes. The tools described are blinded to the sex of the participants whose samples comprise the input dataset. They rely on gene expression data from the protein-coding transcriptome and were not explicitly trained to recognize sex-chromosome-associated genes. No clinical data were included in the training. To our knowledge, gender identity was not recorded or considered in any of the contributing datasets. Furthermore, the data are not disaggregated by sex from the original institutions. A few cancer histotypes identified by clustering are biased (for example, breast cancer) or exclusive to one sex (for example, testicular, ovarian and uterine cancers), and genes on sex chromosomes may play a substantial role in their pathophysiologies (Extended Data Figs. 2 and 3), yet their transcriptional profiles were not the focus of this work. Although the proportions of the sexes have been noted in the clusters annotation, sex differences did not reach significance in clusters discussed in this work and were, thus, not reported.

### KiCS classification review

Occasionally, samples from KiCS have been labeled as ‘concordant in the absence of reference’. In this group, we are counting samples that were assigned to families of tumors close to the target in the absence of a strictly matching subtype. We chose a conservative approach in evaluating these. We counted only those tumors: (1) that matched to a tumor subtype of the expected cell type or tissue of origin to that of the expected diagnosis; (2) where multiple samples with the same diagnosis match the same subtype with the same probability profile; or (3) that match a tumor subtype in which we found reference samples of the same diagnosis but for which there are currently too few samples to create their own class. Finally, each putative match was reviewed by a pediatric oncologist to determine whether the data were sufficient to consider the diagnosis as being confirmed. In total, 16.5% of the tumors were in this category.

### Reporting summary

Further information on research design is available in the Nature Portfolio Reporting Summary linked to this article.

## Online content

Any methods, additional references, Nature Portfolio reporting summaries, source data, extended data, supplementary information, acknowledgements, peer review information; details of author contributions and competing interests; and statements of data and code availability are available at 10.1038/s41591-023-02221-x.

## Supplementary information

Supplementary Information Supplementary figs. 1–17.

Reporting Summary

Supplementary Table Supplementary tables 1–8.

## Data Availability

Expression counts from the Treehouse Childhood Cancer Initiative (including TARGET and TCGA samples) are publicly available (https://treehousegenomics.soe.ucsc.edu/public-data/). Access to raw sequences from GTEx (phs000424.vN.pN) and St. Jude Hospital (https://www.stjude.cloud/) can be requested to their respective institutions. WGS, RNA-seq and methylation data generated as part of the Zero Childhood Cancer Program study are available from the European Genome-phenome Archive (EGA) under accession number EGAS00001004572. The KiCS cohort is available under study number EGAS00001006034. An EGA account is required to download the data.

## Code availability

RACCOON is available as a Python 3 library or can be accessed on GitHub at https://github.com/shlienlab/raccoon, along with documentation. OTTER can be found at https://github.com/shlienlab/otter or at the following website: https://otter.ccm.sickkids.ca.

The corresponding annotation of pediatric tumor types can be found at https://rna-atlas.github.io/.
