# Supplementary material for: Diagnostic classification of childhood cancer using multiscale transcriptomics
Source: Nat Med. 2023 Mar 17;29(3):656–66. doi: 10.1038/s41591-023-02221-x (PMC10033451; doi:10.1038/s41591-023-02221-x)
Supplement: Supplementary file 1 — Supplementary figs. 1–17. [file 41591_2023_2221_MOESM1_ESM.pdf]

nature medicine

Article https://doi.org/10.1038/s41591-023-02221-x

Diagnostic classification of childhood cancer using multiscale transcriptomics

In the format provided by the authors and unedited

Supplemental Figures to Diagnostic classification of childhood cancer using multi-scale transcriptomics

Federico Comitani[1], Joshua O. Nash[1,2], Sarah Cohen-Gogo[3], Astra I. Chang[1], Timmy T. Wen[1], Anant Maheshwari[1], Bipasha Goyal[1], Earvin S. Tio[1], Kevin Tabatabaei[1], Chelsea Mayoh[4,5], Regis Zhao[1], Ben Ho[2,6], Ledia Brunga[1], John E. G. Lawrence[7], Petra Balogh[8], Adrienne M. Flanagan[8,9], Sarah Teichmann[7], Annie Huang[3,6,10], Vijay Ramaswamy[3,10], Johann Hitzler[3,11], Jonathan D. Wasserman[1,3], Rebecca A. Gladdy[12,13,14], Brendan C. Dickson[15], Uri Tabori[1,3,6], Mark J. Cowley[4,5], Sam Behjati[7,16,17], David Malkin[3,10], Anita Villani[3], Meredith S. Irwin[3,10], and Adam Shlien[1,2]

[1]Program in Genetics and Genome Biology, The Hospital for Sick Children, Toronto, Ontario, Canada.

[2]Laboratory of Medicine and Pathobiology, University of Toronto, Toronto, Ontario, Canada.

[3]Department of Paediatrics, The Hospital for Sick Children and University of Toronto, Toronto, Ontario, Canada.

[4]Children's Cancer Institute, Lowy Cancer Research Centre, UNSW Sydney, Sydney, NSW, Australia.

[5]School of Clinical Medicine, UNSW Sydney, Sydney, NSW, Australia.

[6]The Arthur and Sonia Labatt Brain Tumour Research Centre, Hospital for Sick Children, Toronto, Ontario, Canada.

[7]Wellcome Sanger Institute, Hinxton CB10 1SA, UK.

[8]Department of Cellular and Molecular Pathology, Royal National Orthopaedic Hospital, Brockley Hill, Stanmore HA7 4LP, UK.

[9]Research Department of Pathology, University College London Cancer Institute, London WC1E 6DD, UK.

[10]Medical Biophysics, University of Toronto, Toronto, Ontario, Canada.

[11]Program in Developmental and Stem Cell Biology, The Hospital for Sick Children Research Institute, Toronto, ON M5G 0A4, Canada.

[12]Department of Surgical Oncology, Princess Margaret Cancer Centre/Mount Sinai Hospital, Toronto, ON, Canada.

[13]Department of Surgery, University of Toronto, Toronto, ON, Canada.

[14]Lunenfeld-Tanenbaum Research Institute, Sinai Health System, Toronto, Canada.
[15]Department of Pathology and Laboratory Medicine, Mount Sinai Hospital, University of Toronto, Toronto, Canada.
[16]Cambridge University Hospitals NHS Foundation Trust, Cambridge CB2 0QQ, UK.
[17]Department of Paediatrics, University of Cambridge, Cambridge CB2 0QQ, UK.

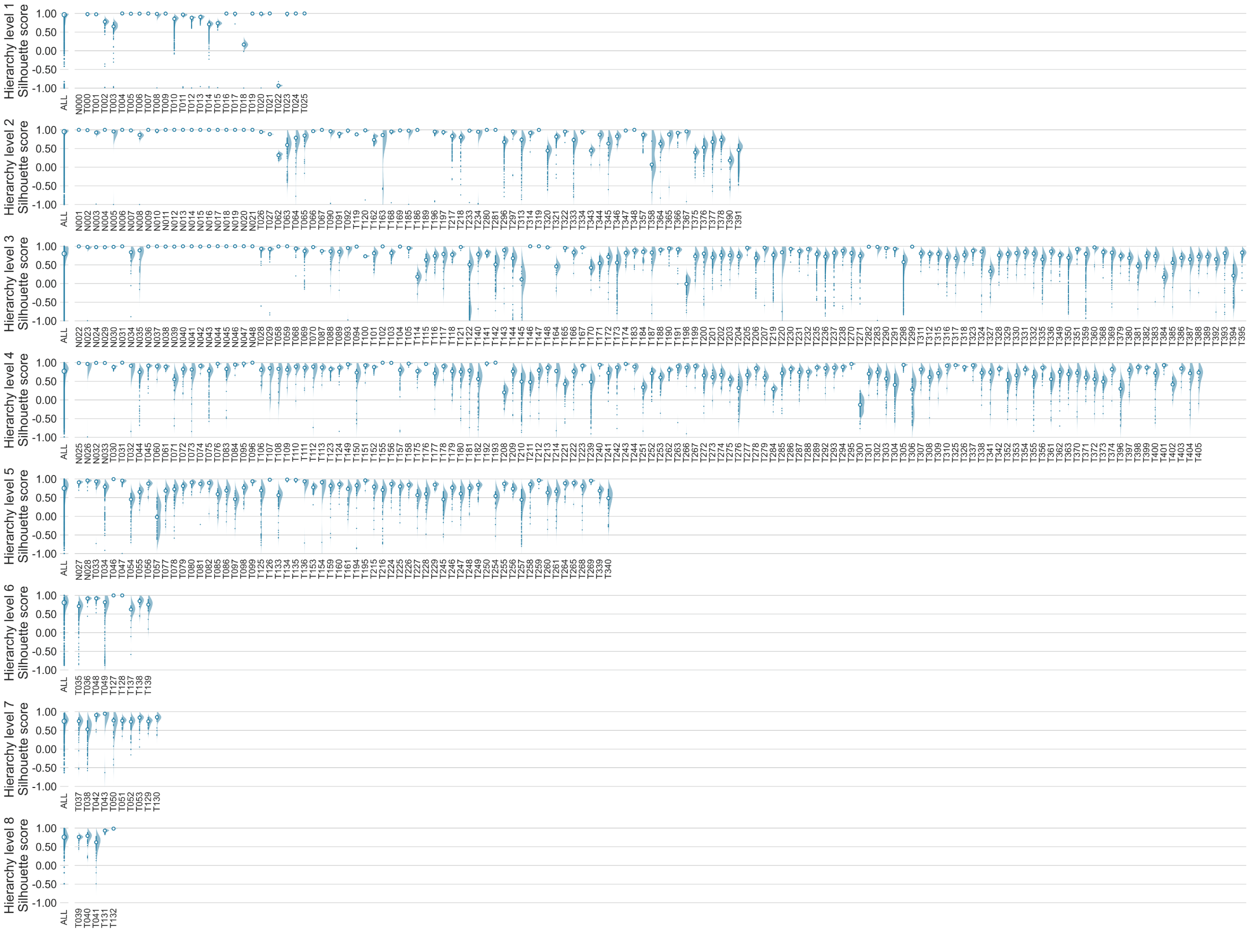

Figure S1: **Silhouette coefficient all tumor classes.** Silhouette coefficients obtained at each point by hierarchical level (rows) and class (columns). Panels on the left contain the values aggregated by level. The scores were adjusted to account for the removal of ribodepleted samples in the final hierarchy. In white circles are the medians for each group. The median score over all points is 0.87, showing high clustering quality across the cohort. The only remarkable exception is T022 Uterine Tumor, found at the first base level of the hierarchy, with a median score of -0.93. It contains both uterine carcinomas and cervical adenocarcinomas which split with high scores at the next iteration. T057 GLI IHDmut noCODEL H3demet (-0.02), and T198 COAD/READ 1 (-0.01) and T300 BRCA LUMA+NORM ILC (-0.13) also have negative values, but close to zero. Their sibling nodes have positive (¿0.28) silhouette scores, suggesting there may not be much overlap, but rather these classes contain samples of difficult assignment.

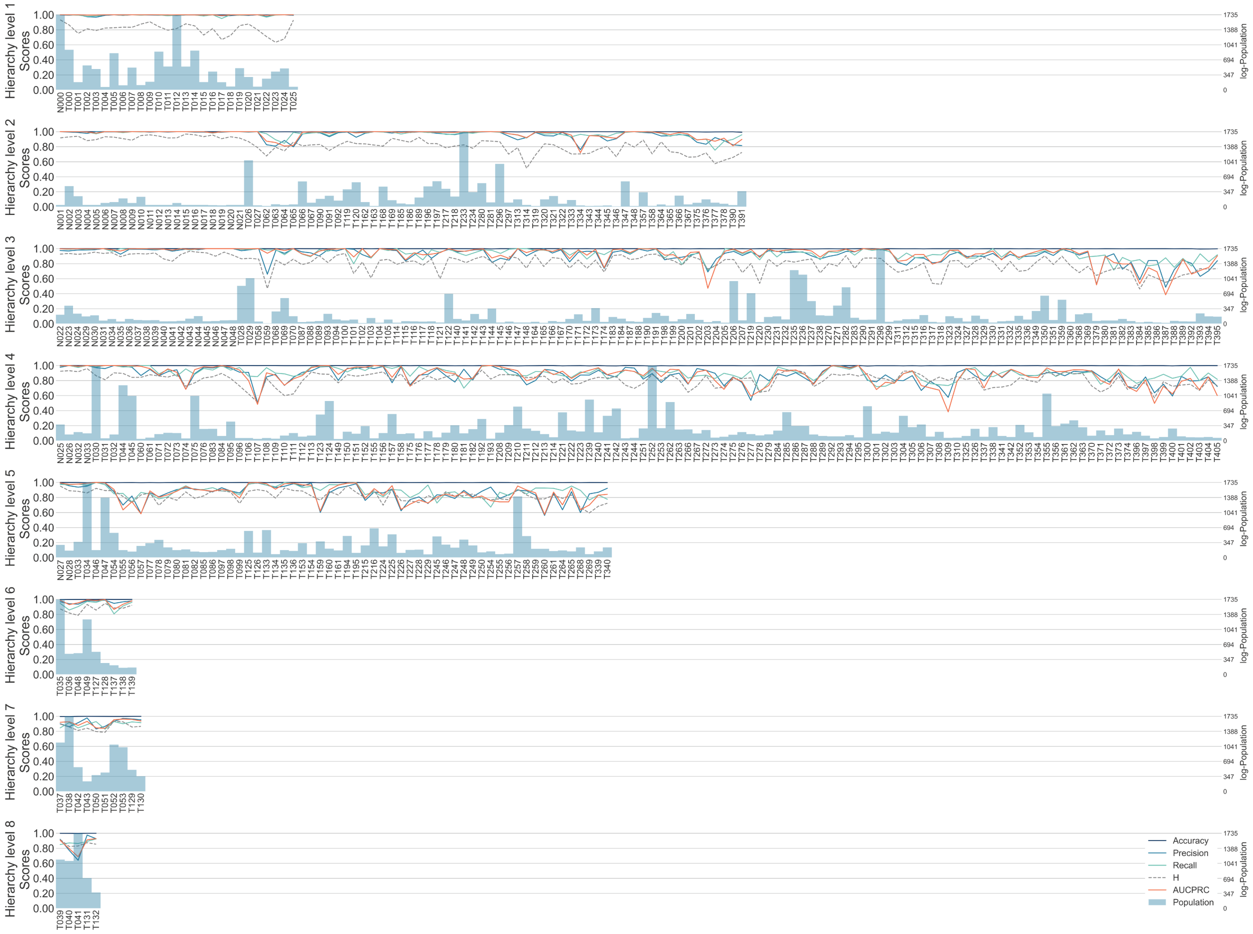

Figure S2: **Classifier scores all classes.** Validation scores obtained by the randomized 5x5-fold validation, on all available classes stratified by hierarchy level. These include accuracy (dark blue), area under the curve precision-recall (AUCPR, orange), precision (blue) recall (green) and hierarchical similarity H (dashed grey). All averaged scores are calculated as micro (m) averages. The total reference population of each class is also shown as shaded bars (blue). Median MAUCPR of 0.95 with only a handful of classes falling below 0.5, four non-pediatric solid tumor subtypes and a subtype of TGCT at intermediate maturation (T107). 47 fall below 0.75 AUCPR.

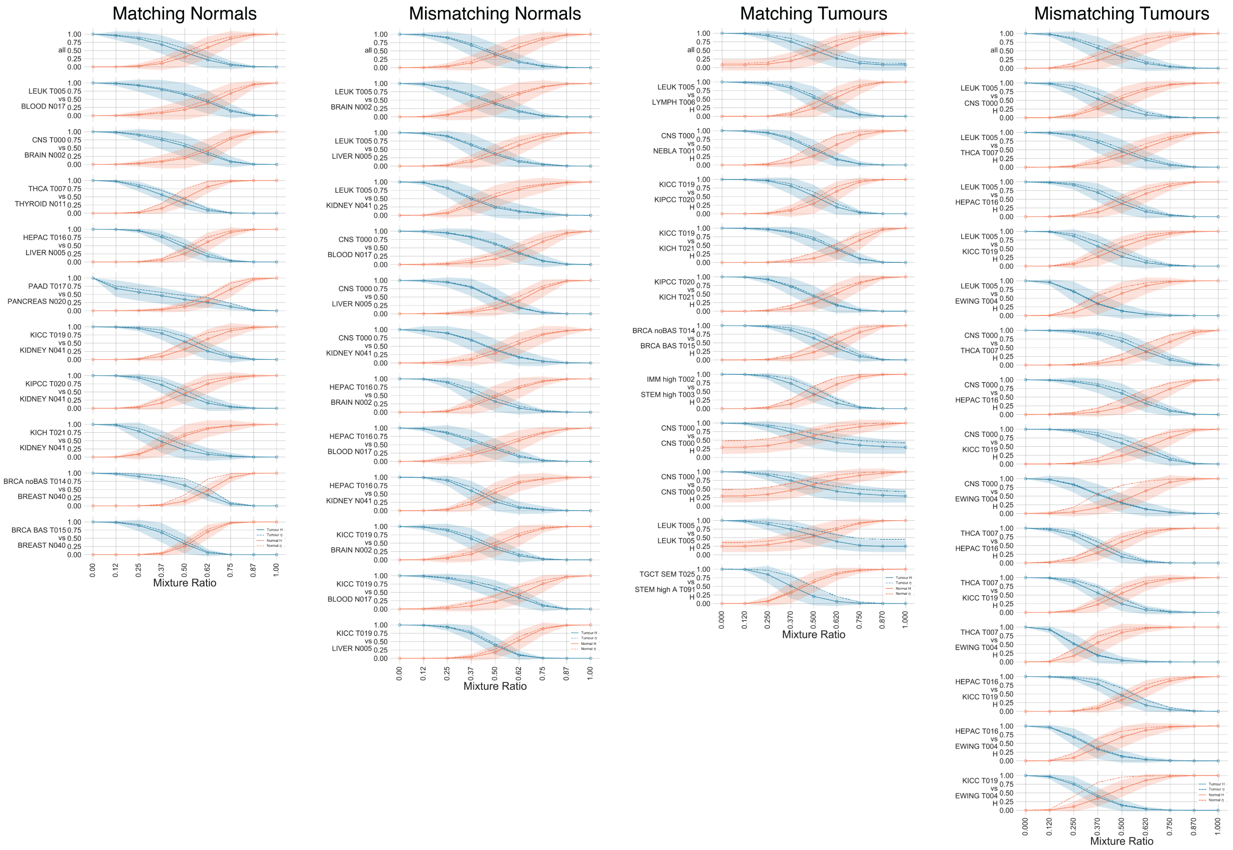

Figure S3: **Classifier benchmark: tumor-normal mixtures.** Consistency of classification quantified by Hierarchical similarity scores (H as full lines and h as dashed lines) when mixing the expression profiles of two samples at different ratios. Samples are taken from different classes with curves representing the consistency of prediction when using the first (blue) or the second (orange) unmixed sample as ground truth. The experiments were repeated for tumors mixed with matched normal tissues (e.g. tumor and normal from the same organ), mismatched normal tissues (from different organs), matched and mismatched tumors. Values are shown as means and error bars are standard deviations calculated over up to 100 samples per class and 500 randomized iterations. Signal from matching normal tissue is identified at low probabilities (average H = .03) when at least 25% of its gene expression is present, while tumor type calling is marginally affected (avg .86). Confidence values are still higher for tumor components at a 50/50 mixture (= .44 for tumor, .30 for normal tissue). Considerably more variability in the results is observed when tissues from non-matching organs are introduced in the mixture. Per tumor-type differences are observed across all experiments. OTTER is less confident with thyroid carcinoma, while Ewing sarcoma is recognized at much lower mixing percentages. Both are minority classes, suggesting the transcriptional characterization, rather than poor training is at the root of these differences. Overall, kidney subtypes do not mix well; the model shows a preference to call renal clear cell carcinomas and papillary cell carcinomas over kidney chromophores. η curves have overall higher values than the corresponding H; the classifier prefers thus calling both mixture components, rather than lower their probabilities.

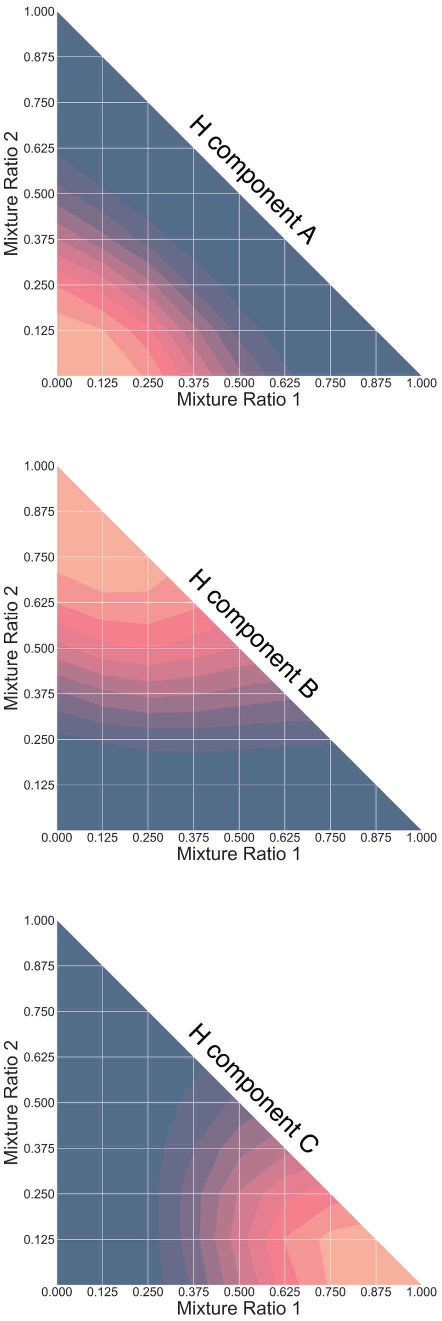

Figure S4: **Classifier benchmark: 3 samples mixtures.** Consistency of classification measured by the hierarchical similarity score when mixing expression profiles of three samples at different ratios. The plots show the measure when using the first (top), the second (center) and the third (bottom) sample original prediction as aground truth. The mixture was composed of leukemias, CNS and kidney samples.100 samples from each group were extracted and 500 triplets' permutations were mixed at set ratios. In the contour plots, each component starts to be identified by the classifier results at about .3 of ratio and quickly rises to its maximum of 1 with more than .75.

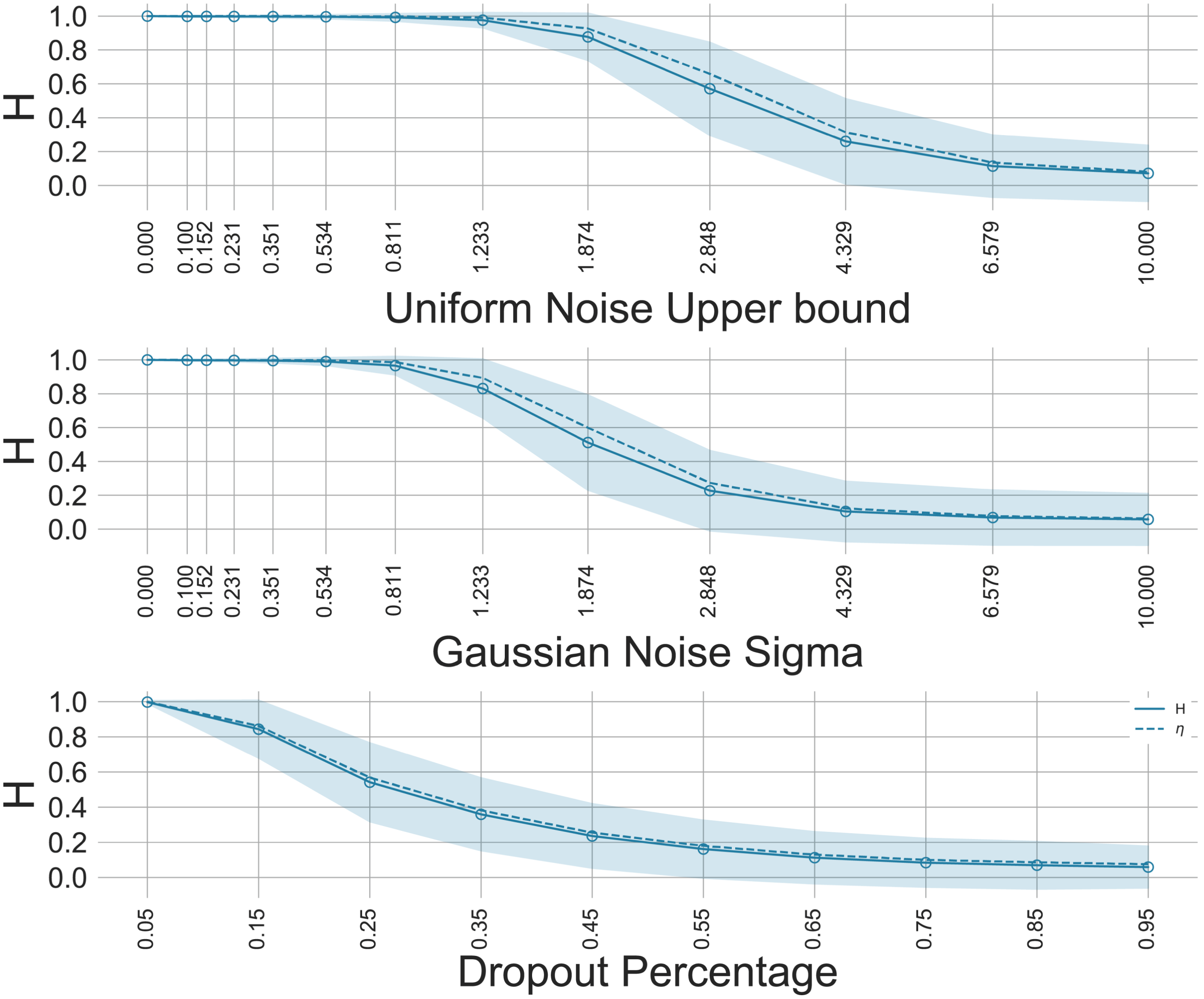

Figure S5: **Classifier benchmark: noise.** Limit of detection tests for OTTER. The consistency of classification results on a test set of samples is measured as a function of increasing synthetic noise. The ground truth was set as the original classification without noise. Values are shown as means and error bars are standard deviations calculated over 500 samples and 5 randomized iterations. The level noise applied to each gene is taken from a uniform (top) or gaussian (center) distribution with different parameters' values (upper bound and s respectively). With a uniform noise distribution, we observe a steady decrease in performance between 1.2 and 6.6 TPM above which the expression patterns characterizing each class are lost almost completely. The Gaussian sigma threshold is lower with losses observed starting at 1.23 and down to 4.3 TPM. 66.8% of the added stochastic noise falls under the Gaussian s, while tails extend beyond the set uniform distribution boundaries and are thinner, thus the presence of fewer yet highly noisy genes have a stronger impact on the classification quality than equally distributed noise across all genes. The bottom plot shows the effect of dropout, where the expression percentage of randomly selected genes was "deactivated" to simulate corruption of the input. Dropping just 25% of the genes can lead to a loss of 40% of H on average; in most cases the classes will be still called, but with a loss in confidence and lacking the ability to discern subtypes. This effect is strongly dependent on which genes are affected, as reflected in the large standard deviation (above .2 H).

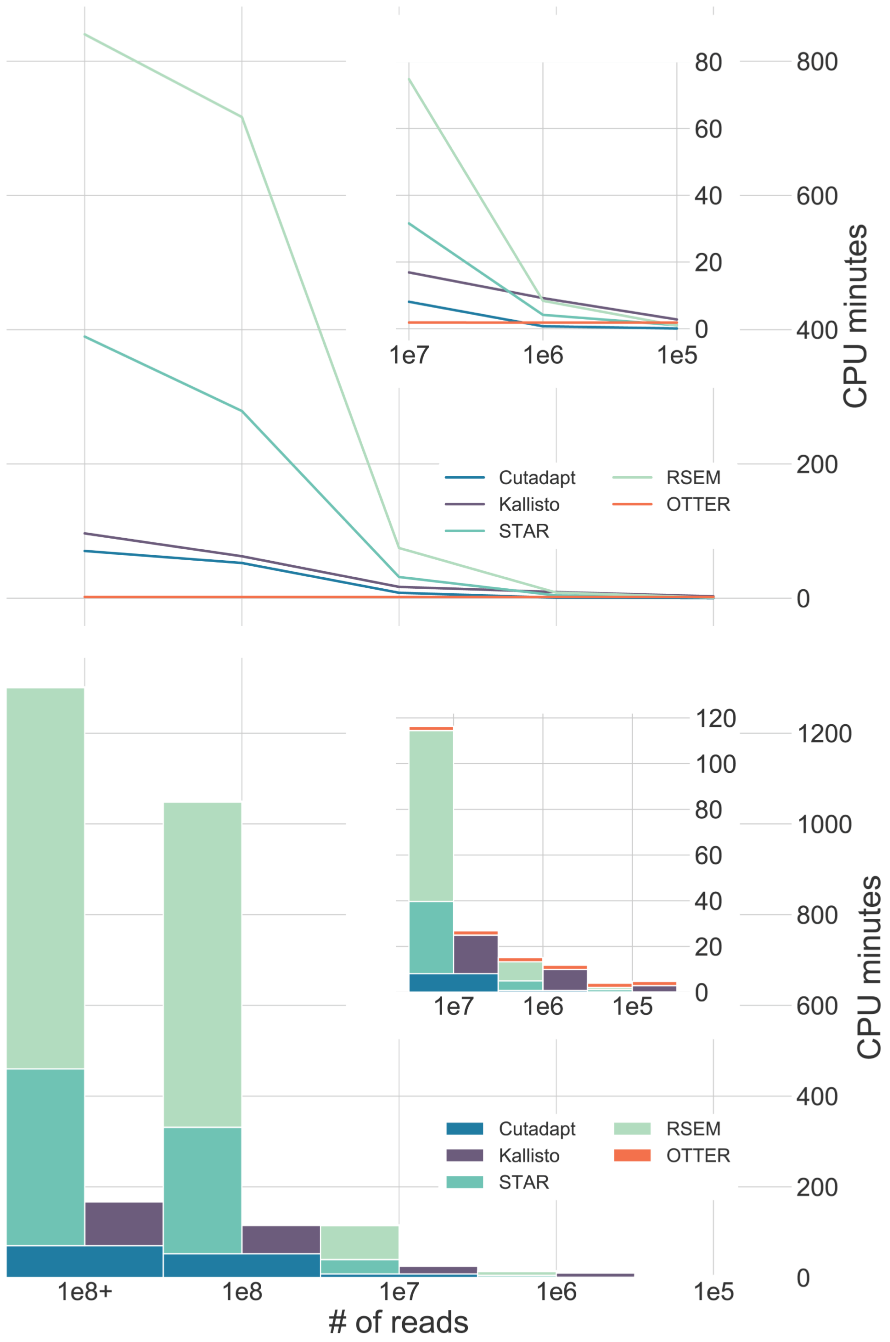

Figure S6: **Classifier benchmark: pipeline times.** Computation times for the full toil-RNASeq pipeline starting from fastq files and including the classification. At the top, each step is separate, and at the bottom two possible branches are shown as separate staked bars (STAR+RSEM left and Kallisto right). Insets focus on samples with 1 million reads. These include Cutadapt (blue), common to both branches, Kallisto (purple), STAR (dark green), RSEM (light green) and OTTER (orange). The Kallisto version of the toil-RNASeq pipeline is faster, yet less accurate. Starting from fastq files with 108 reads, the results are obtained in less than 2 CPU hours. With 1 million read, 10 to 15 minutes are required for both Kallisto and RSEM. Benchmarks were run on our local cluster, equipped with Xeon E5-2670 v2 @ 2.50GHz and Xeon Gold 6140 CPU @ 2.30GHz CPUs.

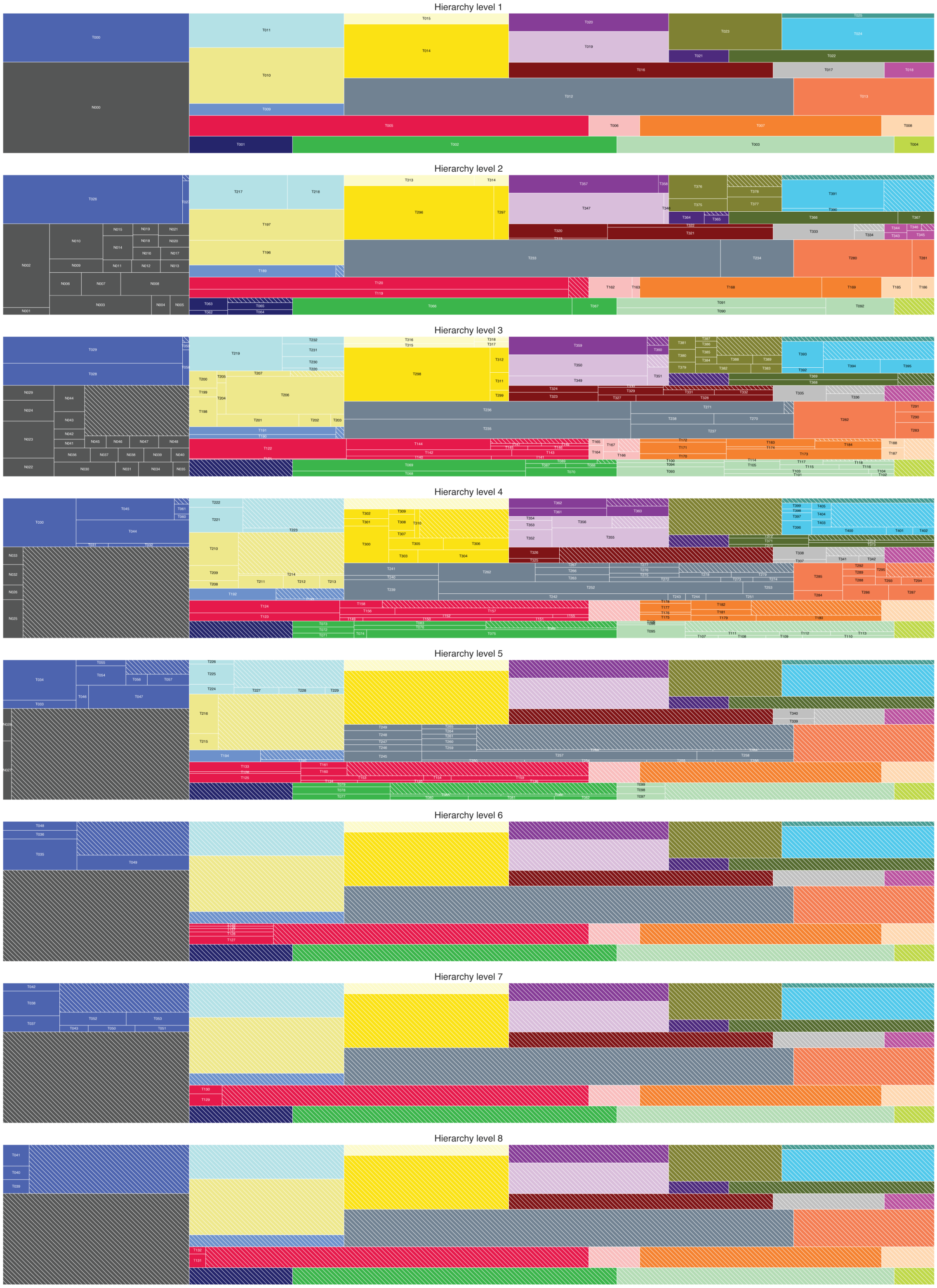

Figure S7: **Transcriptional atlas treemap.** Treemaps of the separate hierarchical levels of the transcriptional atlas. The size of each rectangle, representing one class, is proportional to its population. Colors represent separate families, hatching covers leftover areas from the prior level of the hierarchy.

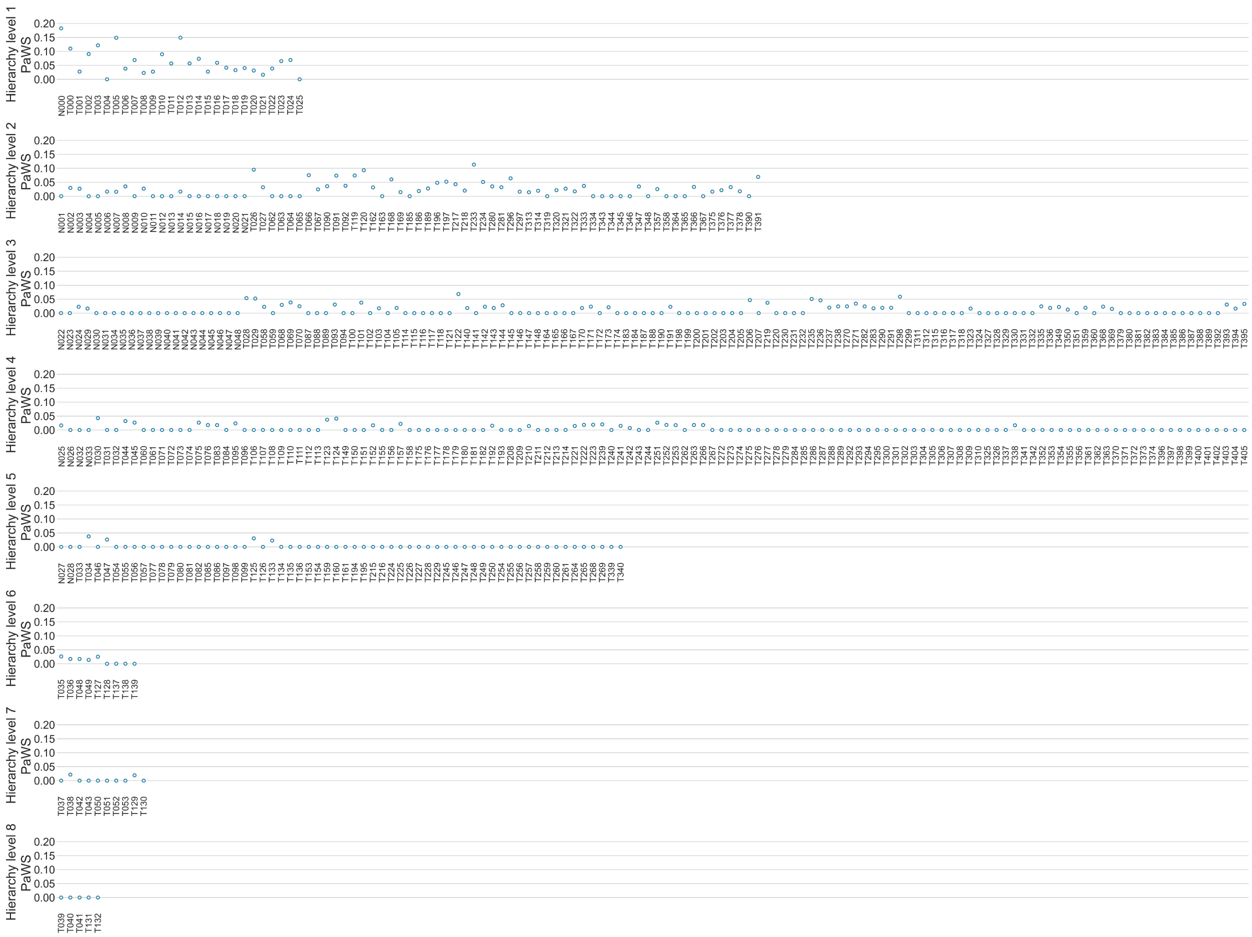

Figure S8: **PaWS of all classes.** Population weighted splits score for all classes (columns), stratified by hierarchical level (rows).

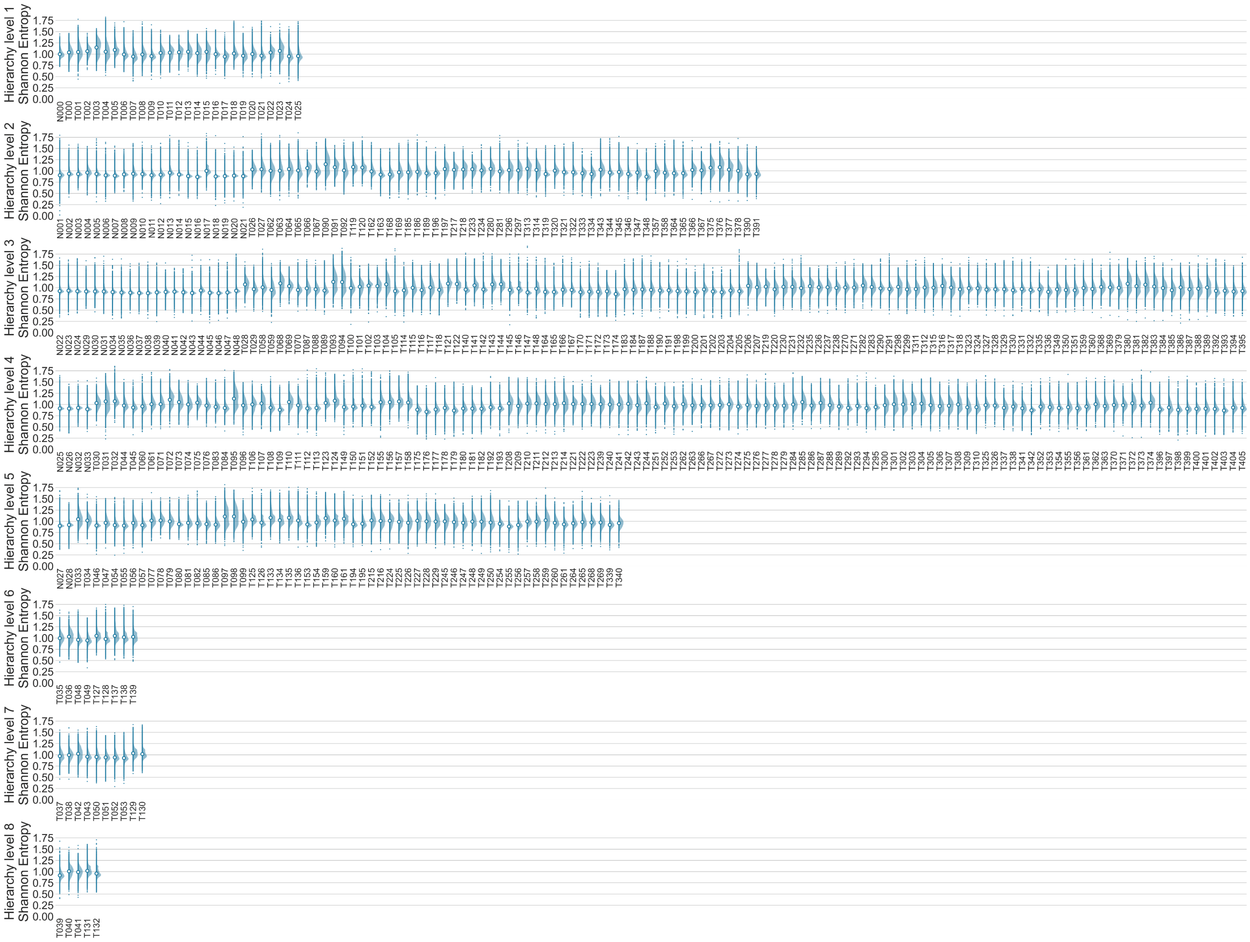

Figure S9: **Entropy of all classes.** Shannon Entropy for all classes (columns), stratified by hierarchical level (rows).

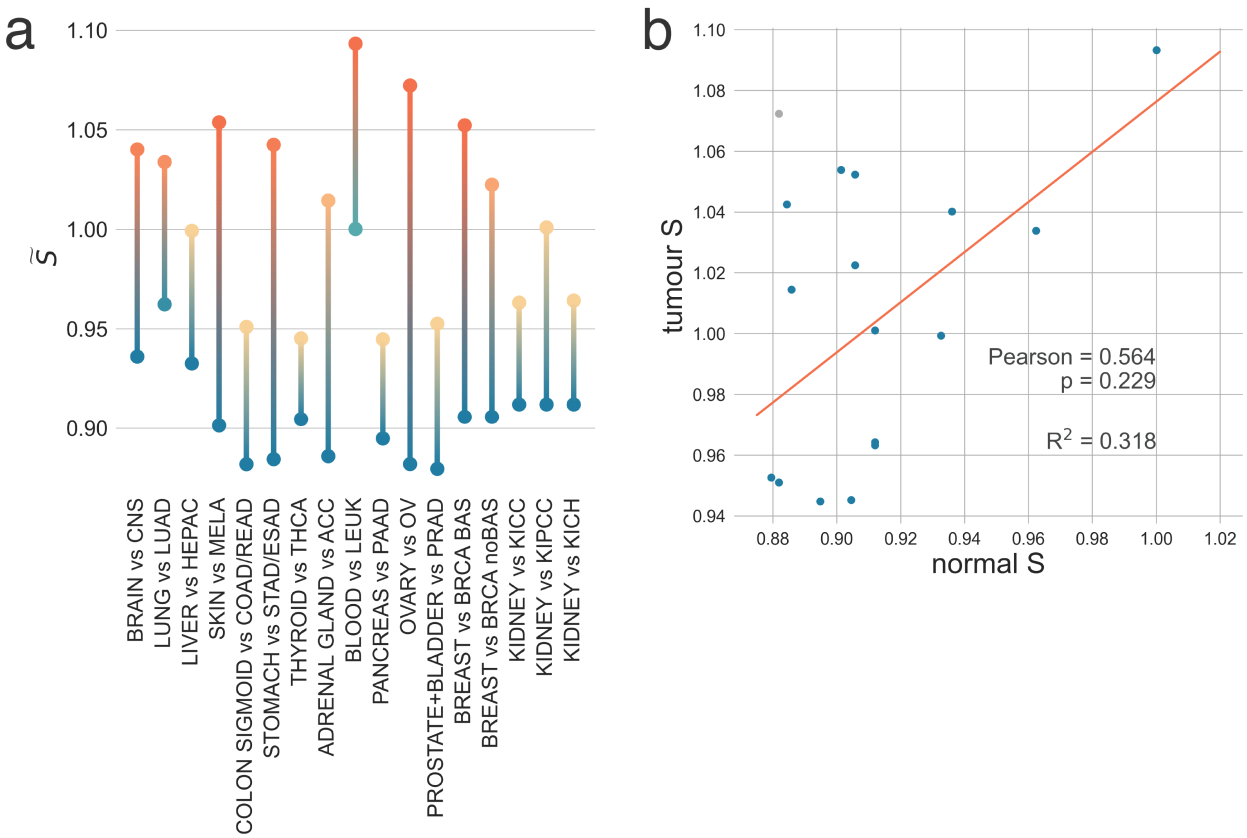

Figure S10: **Entropy dependence on tissue of origin.** A) Comparison of median entropy values between tumor and normal tissue samples for different organs. Values for normal tissues are shown in shades of blue, values for malignant samples are shown in shades of orange. B) Scatter plot of normal vs tumoral entropy for the selected tissue types. Pearson's correlation and the coefficient of determination from a linear fit (red) are shown. The grey dot corresponds to the value for ovarian cystadenocarcinoma and ovary tissue. The ratio of tumor over normal entropy for this data point lies at ¿ 2.15 s and was thus considered an outlier and excluded from the linear regression and correlation calculations.

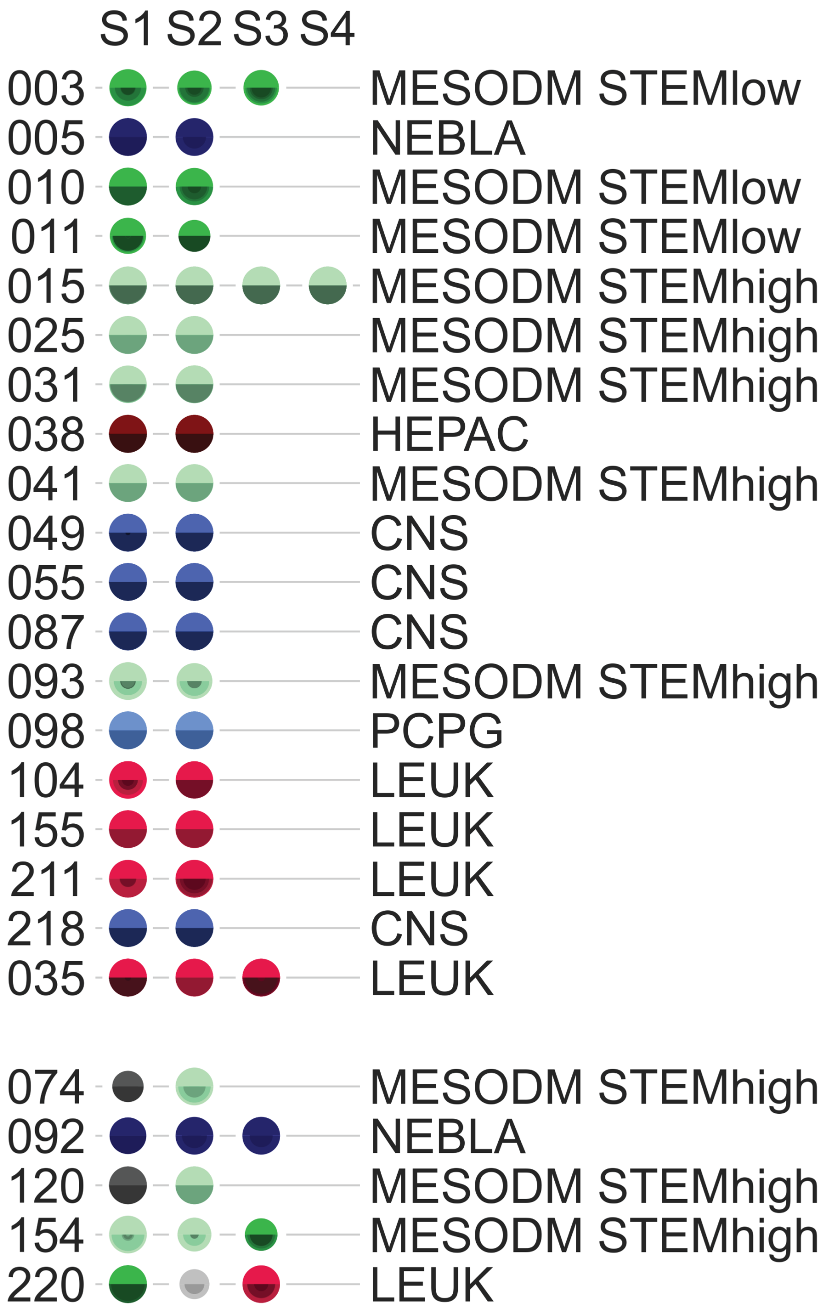

Figure S11: **Multi-time samples complete.** Majority class assignments for patients with samples taken at multiple time points. Each sample is shown as a dot, with size proportional to the class probability. The full circle represents the majority class at the first hierarchical level, and the bottom half circles in transparency show further subtypes. On the right, the name of the transcriptional family assigned to the first sample is shown in short form, except for those where normal contamination was dominant, in which case the next available sample is used. This version includes two patients who developed two separate primaries, and that were removed from Fig 6c.

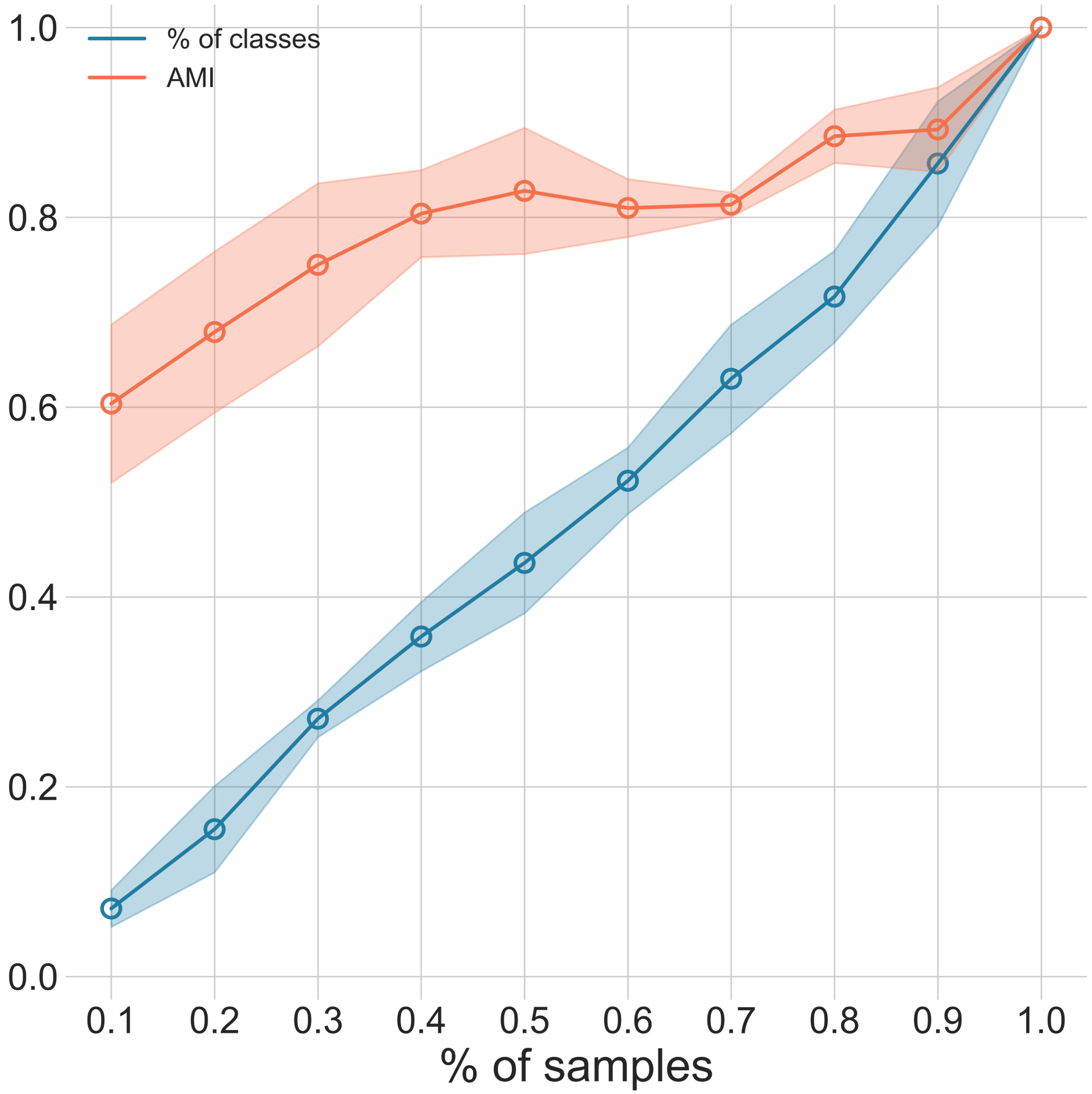

Figure S12: **Clustering robustness test.** Adjusted Mutual Information (AMI, in orange), and percentage of classes identified (in blue) when running RACCOON on random subsets of the CNS tumors class (total n=927). The results obtained on the full dataset are set as ground truth for both measures. For each 10 percent of population reduction, the random subsampling was repeated five times. Mean AMI at different percentages over 5 randomized iterations are shown as circles, the standard deviation ranges are shown as colored areas. AMI ranges from 0 (different partitions) to 1 (equivalent partitions) and is defined for equal population groups and flat partitions. The hierarchy was flattened, reassigning samples to maximize AMI and comparison was limited to common samples. RACCOON parameters were fixed across experiments. The class numbers reduction is linear in the population percentage The mean AMI is stable at .89 when only 80% of the data is used and decreases to .60 when only 10% of the samples are kept.

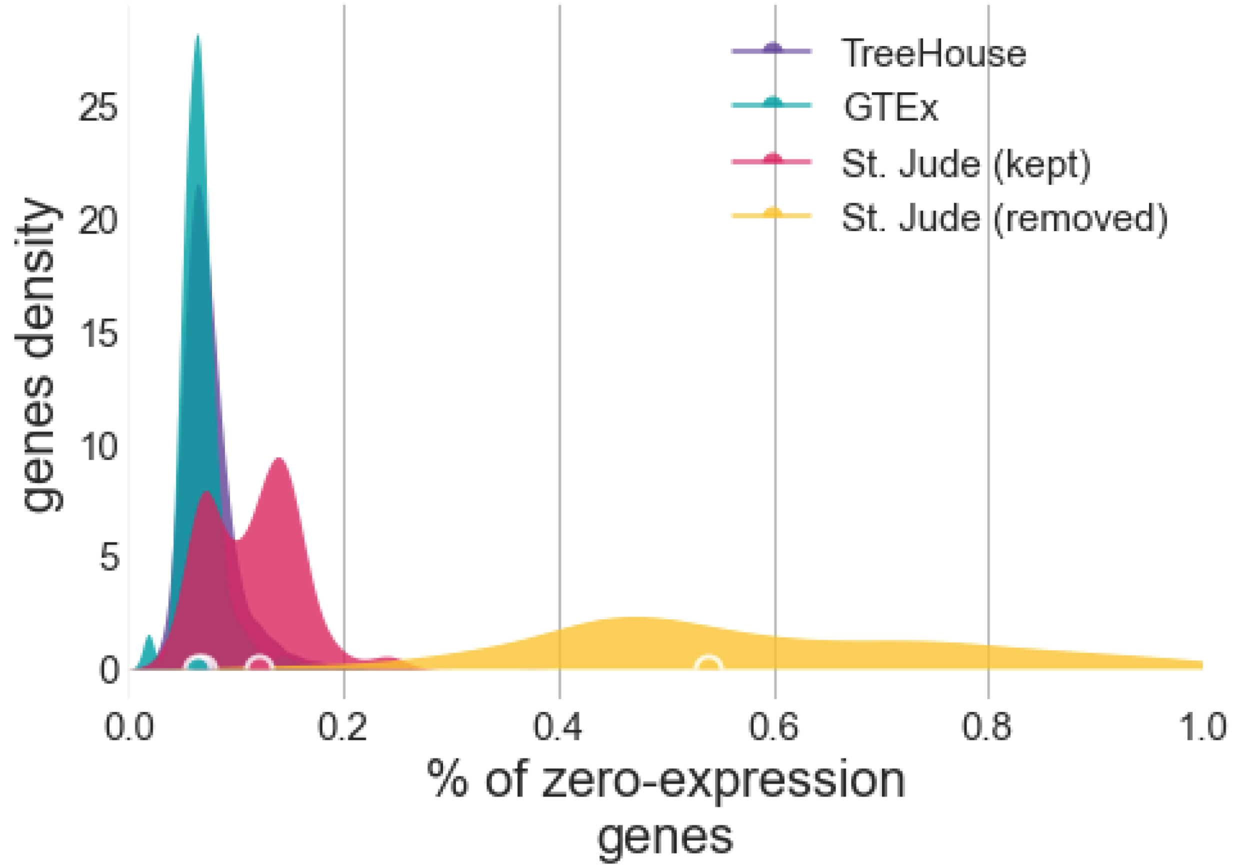

Figure S13: **Expression distribution QC.** Percentage distribution of genes with zero expression for samples in different cohorts: Treehouse (purple), GTEx (cyan), St. Jude PGCP included in our study (magenta), and St. Jude PGCP excluded due to a low number of expressed genes (yellow).

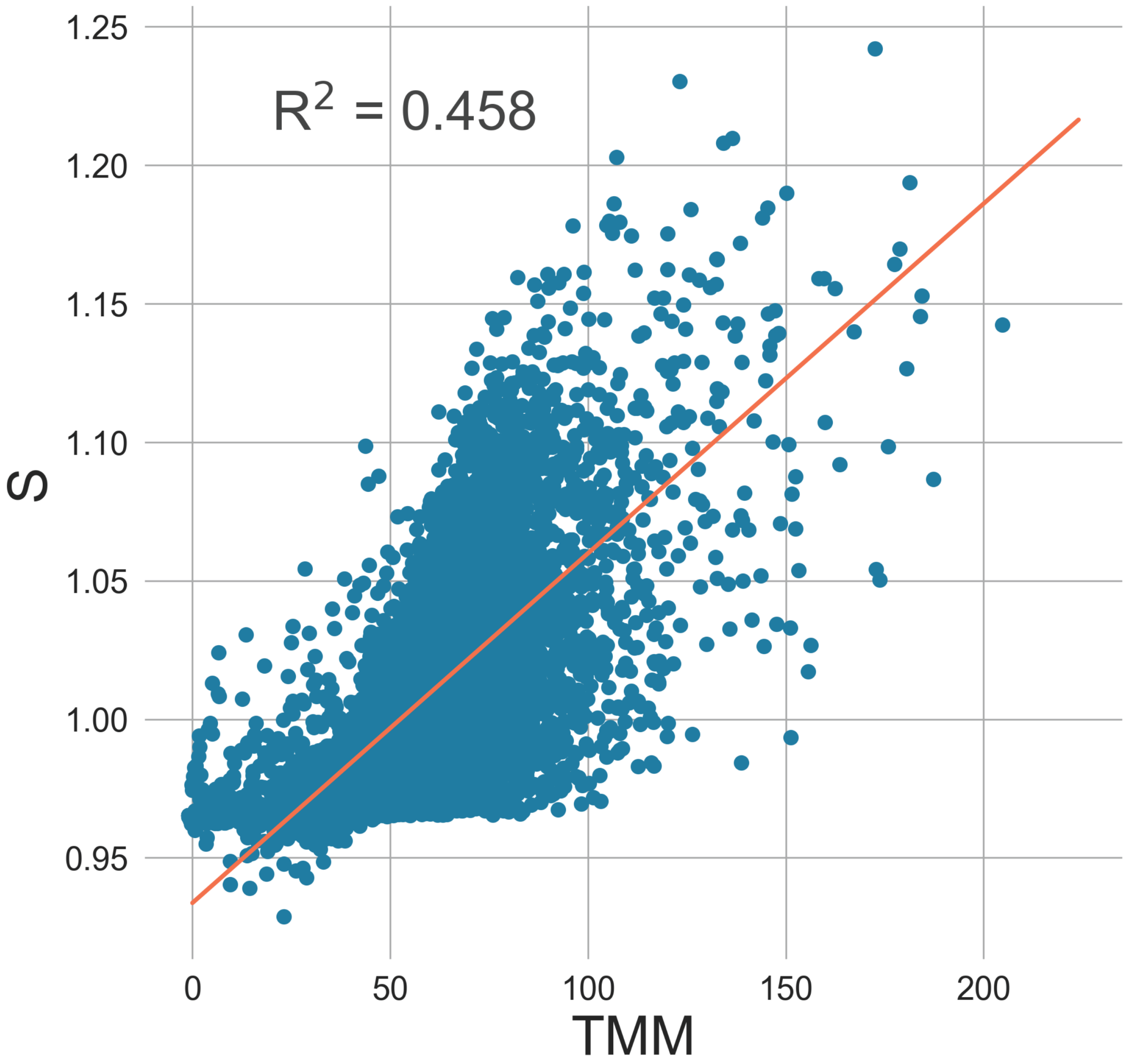

Figure S14: **Adjusted entropy.** Scatter plot showing the original entropy values per class as a function of their mean expression of each gene (in TMM). In red is the linear regression, whose coefficient of determination is shown.

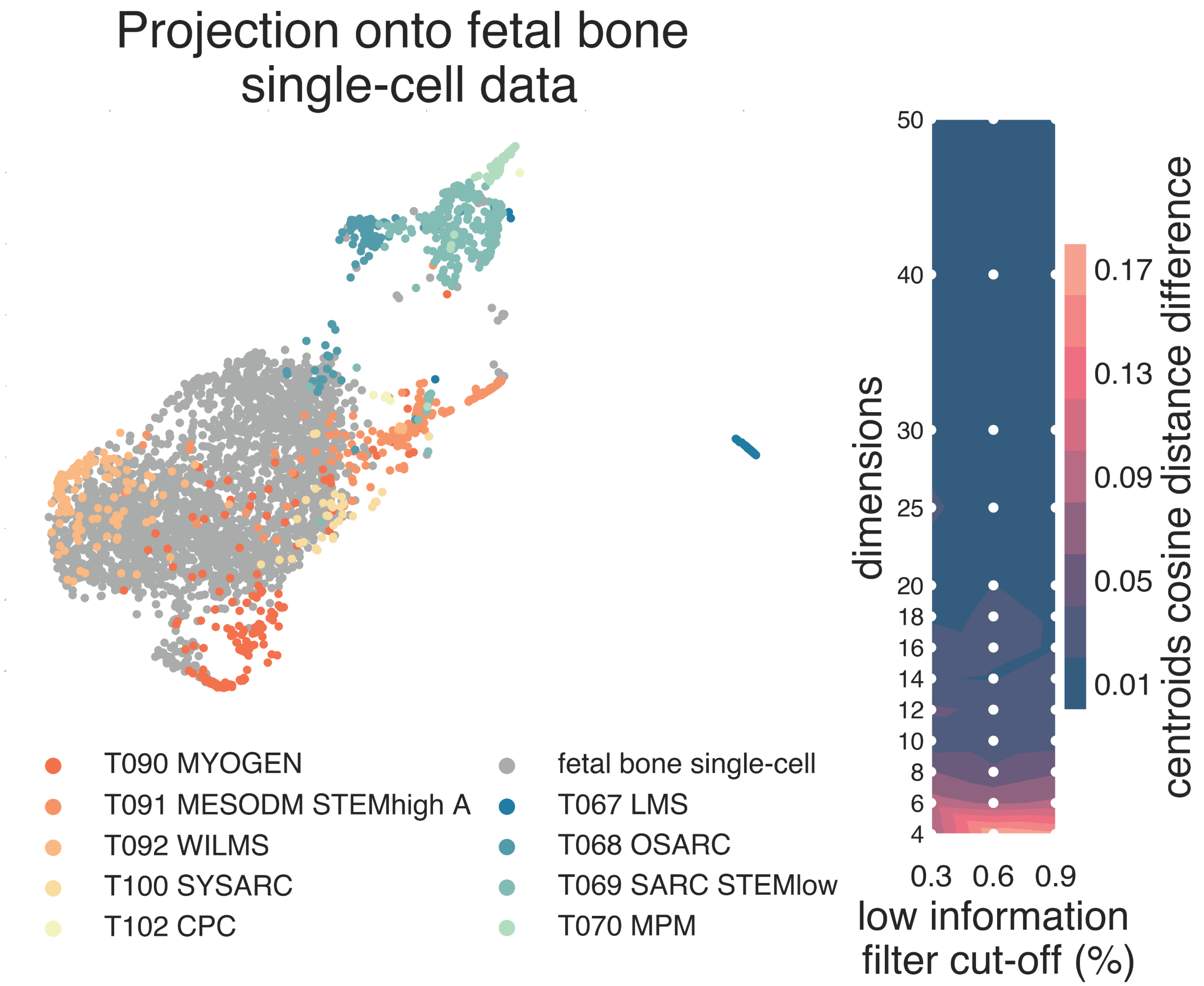

Figure S15: **Sarcomas projection onto single-cell data.** A) 2-dimension UMAP projection of mesodermal tumors T002 in shades of blue and green, and T003 in shades of orange. This data was projected onto a map trained on expression data from single-cell data from fetal bone tissue (in grey). B) The cosine centroids distance difference between T002 and T003 and the single cell data measured on projections with different parameters. As UMAP does not conserve distances, but rather the nearest-neighbors relationship within points, different target dimensions, and low-information filter cutoffs were explored with a grid search to test the robustness of the centroids' distances comparison across parametrizations. The value is strictly positive, supporting the idea that T002 is farther away from the fetal tissue than T003, independently of artifacts that could be introduced by the projection and its choice of parameters. As a reference, the cosine distance difference with the full 16,452 genes, common to bulk and the single cell datasets, is ¿ 0.01.

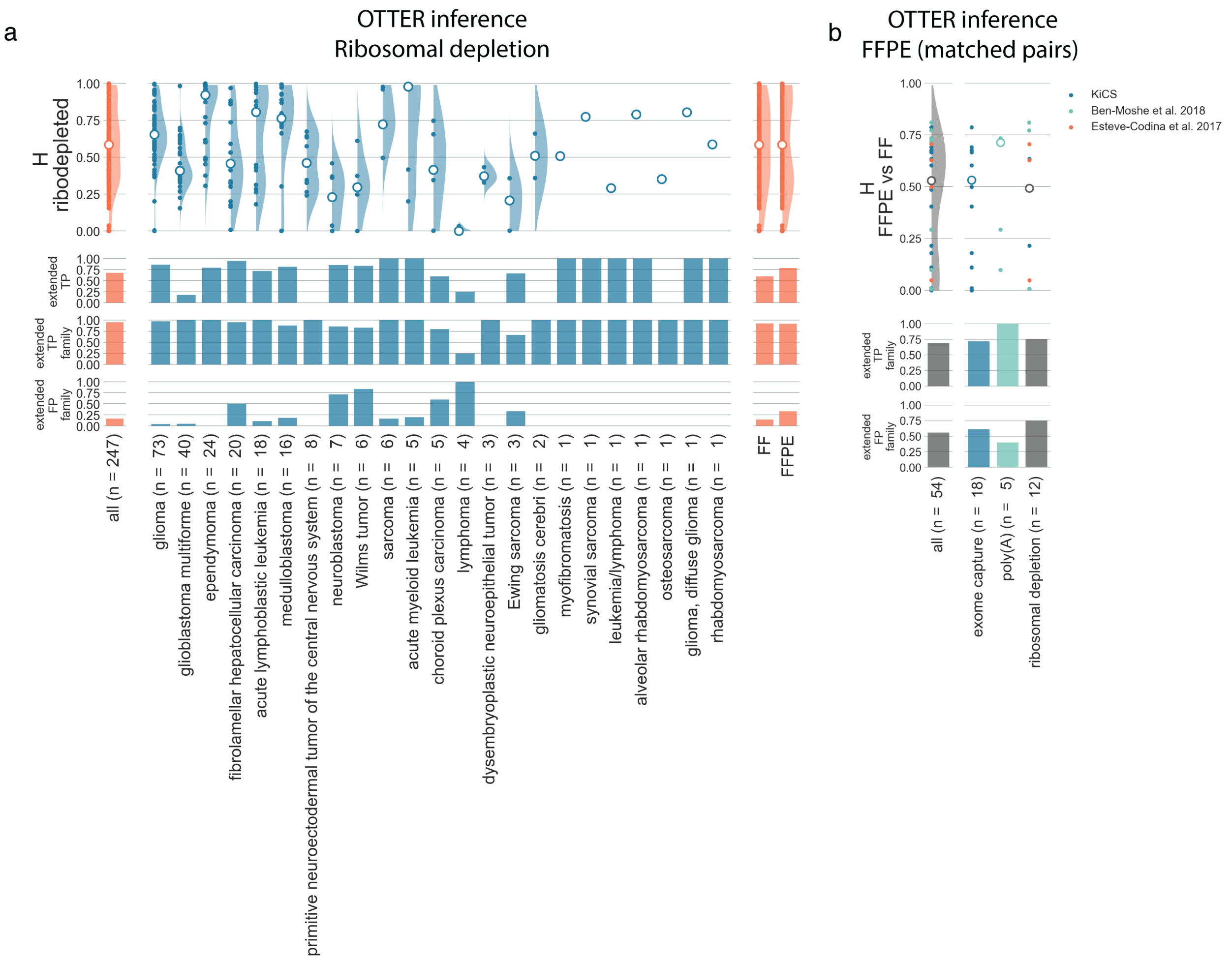

Figure S16: **Library preparation and preservation benchmarks.** A) OTTER prediction performance obtained with 247 samples treated with ribosomal depletion. From the top are the distribution of hierarchical similarities and their median, true positives (with a confidence threshold set at 5%) matching the exact tumor type, true positives to the tumor family, and false positives matching other tumor families. Results are shown for the whole cohort (left, orange), aggregated by tumor type (center, blue) and by storage method (left, orange). The classifier can identify the correct tumor type for most cases at reduced (0.585 median H) and there is considerable variability among tumor types. For 95% of cases, the tumor is matched to the expected family of cancers with ¿5% confidence. For 68% of cases, the tumor is matched to the expected subtype. The false positive rate is 16% (tumor mapping to a different tumor family at ¿ 5%L), it is a conservative estimate, given the uncertainty of the original labels. 93 out of 247 samples were stored as formalin-fixed paraffin-embedded (FFPE, right panel, in orange). Yet, we observe no significant difference in the performance between FFPE and FF samples, but the false positives percentage is higher in FFPE samples (33%). B) Performance obtained with 52 FFPE samples paired to FF samples. From the top are the distribution of hierarchical similarities and their median, true positives (with a confidence threshold set at 5%) matching the exact tumor type, true positives to the tumor family, and false positives matching other tumor families. Results are shown for the whole cohort (left, black) and aggregated by library preparation technique, exome capture (left), poly-adenylation, and ribosomal depletion. Different cohorts are shown with different colors. The median H is 0.53, and 69% percent of the samples were matched to the expected family. Again, the critical difference with FFPE samples is a higher false positive rate (56%). We then stratified the FFPE tumors by library preparation to demonstrate that the effects are additive.

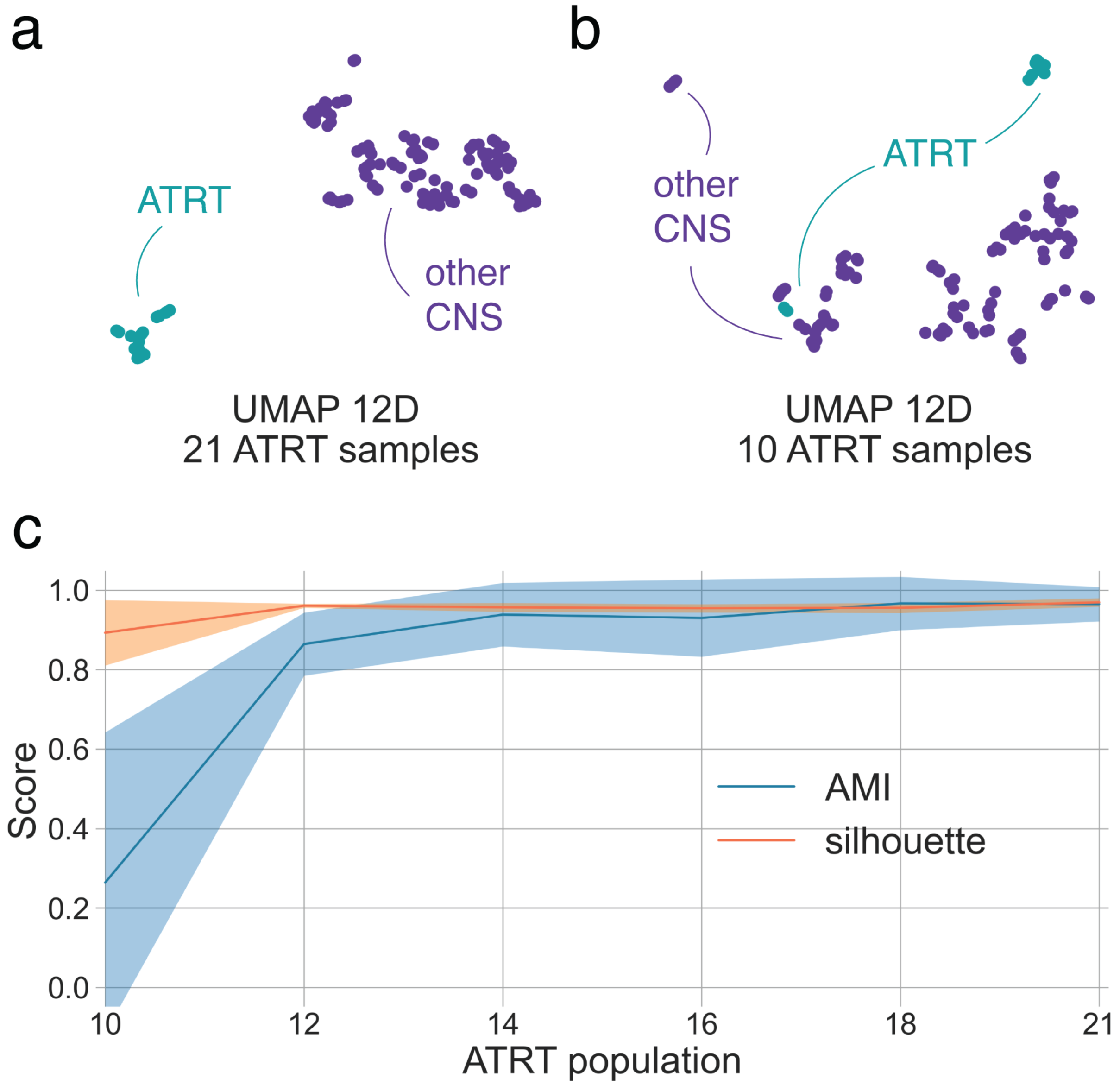

Figure S17: **Extending the tumor atlas, ATRT.** A) 2d UMAP projection of 21 ATRT samples and a subset of 100 mixed high-grade gliomas from T043. B) 2d UMAP projection of a subset of 10 ATRT samples and 100 mixed high-grade gliomas from T043. C) Adjusted mutual information (AMI, in blue) and silhouette score (in orange) comparing randomized runs of RACCOON with different sets of high-grade gliomas and ATRTs. The ground truth for AMI was set as a complete separation between ATRT and all other samples. The experiment was repeated with an increasing population of ATRT. Lines are the average of 5 repetitions with randomized subsets, shades show their standard deviation. The threshold for creating an ATRT-specific class is 12 tumors (mean AMI¿.8). With 10 tumors only 1/5 of the randomized iterations yielded an ATRT-only class (mean AMI = 0.26).The silhouette score was above .8 in all cases, supporting the reliability of the clustering result in all iterations. These results support the idea as new samples are added to the next iterations of the transcriptional atlas, currently, missing subtypes could be identified by RACCOON with as little as 12 samples, depending on the tumor type.
